# Does climatic variation drive the adjustment of functional traits? An assessment of Tropical Montane Cloud Forest tree species

**DOI:** 10.3389/fpls.2025.1555607

**Published:** 2025-06-04

**Authors:** Agustina Rosa Andrés-Hernández, Ernesto C. Rodríguez-Ramírez

**Affiliations:** ^1^ Facultad de Ciencias Biológicas, Benemérita Universidad Autónoma de Puebla, Puebla, Pue, Mexico; ^2^ Laboratorio de Dendrocronología, Universidad Continental, Huancayo, Junín, Peru

**Keywords:** climate variability, drought stress, endangered species acclimation, environmental adaptation, wood anatomy, leaf morphological traits

## Abstract

**Introduction:**

Tropical montane cloud forests (TMCFs) host specialized plant species reliant on persistent atmospheric humidity, including fog immersion obligates and relict assemblages. Understanding anatomical and morphological adaptations in TMCF woody angiosperms is critical for elucidating their acclimation strategies to hydric stress under shifting fog regimes. This study investigates interspecific variability in wood and leaf traits among 10 TMCF tree species in Mexico’s Medio Monte Natural Protected Area, hypothesizing that distinct anatomical strategies emerge in response to climatic stressors.

**Methods:**

Wood anatomical (e.g., vessel density, hydraulic diameter, fiber length) and leaf morphological traits (e.g., lamina length, vein density, leaf organization) were analyzed across species. Traits were correlated with climatic variables—mean maximum/minimum temperatures, monthly precipitation, and evapotranspiration—to identify adaptive patterns. Statistical analyses quantified interspecific differences and assessed trait-climate relationships.

**Results:**

Significant interspecific divergence occurred in both wood and leaf traits. Wood anatomy was strongly influenced by mean maximum temperature, precipitation, and evapotranspiration, affecting vessel density, vulnerability index, ray dimensions, and fiber length. Leaf traits correlated with temperature extremes and evapotranspiration, driving variation in leaf size, apex/base morphology, venation complexity, and marginal teeth. Notably, hydraulic efficiency (e.g., wider vessels) aligned with higher precipitation, while drought-associated traits (e.g., denser veins) linked to elevated temperatures.

**Discussion:**

TMCF species exhibit trait-based strategies balancing hydraulic safety and efficiency, reflecting niche partitioning under microclimatic gradients. Temperature and water availability differentially shape wood and leaf adaptations, with vessel architecture and venation patterns acting as key regulators of water loss. These findings underscore the functional diversity of TMCF trees and their capacity to acclimate to environmental variability. Conservation efforts must prioritize microclimate preservation to safeguard these adaptive traits amid climate change.

## 1 Introduction

Tropical Montane Cloud Forests (TMCFs; *sensu* Bruijnzeel et al., 2010) exhibit substantial biodiversity and contribute significantly to essential ecosystem services in hotspot mountainous areas (Gentry, 1992; Fahey et al., 2016; Richter et al., 2024). The adaptability of TMCF plants to hydric gradients is determined by the frequency of fog, mist and potential evapotranspiration (Bruijnzeel et al., 2011; Crausbay et al., 2014). Along this gradient, tree height, density, and forest species diversity are progressively reduced and morpho-physiological acclimation and/or adaptations to resist or avoid hydric stress are more common (Thybring and Fredriksson, 2021; Rodríguez-Ramírez et al., 2022; López-Calvillo et al., 2023). Nonetheless, the occurrence of longer than typical hydric stress seasons or droughts over an extended number of years in TMCFs can result in reduced plant growth, seedling mortality, and large-scale tree mortality (Choat et al., 2018; Rodríguez‐Ramírez et al., 2024a). Climate change poses a significant potential risk to the future survival of TMCFs (Los et al., 2021; Ramírez-Barahona et al., 2025). Projected climate scenarios predict a temperature increase of 4.1-5°C above current levels and an increase in CO_2_ emissions to 70.04 gigatons for Shared Socioeconomic Pathways (SSP) 3-7.0 and 116.8 gigatons for SSP 5-8.5 (Taylor et al., 2012).

Typically, hydric stress on plants is studied in controlled greenhouses; however, this approach does not consider the complexity of TMCF systems and the responses of relict endemic trees (Eller et al., 2020). Thus, a mechanistic understanding of how TMCF wetness variation determines community composition and function is required in the field to predict TMCF responses to climate change (Andrés-Hernández et al., 2023). Functional traits have often been defined as morphological, physiological and phenological plant characteristics that influence the success of a genotype (individual), which are in turn influenced by environmental interactions and evolutionary processes, environmental interactions and evolutionary processes (Anderegg and Meinzer, 2015; Tng et al., 2018). New evidence about trait variation in plants, such as leaf vein acclimation (Eller et al., 2020), wood anatomy (Argüelles-Marrón et al., 2023) and seed size/mast (Burns, 2012) convinced ecologists that functional traits are the best approach to achieving predictive understanding (McGill et al., 2006; Sobral, 2021; Andrés-Hernández et al., 2023).

In this context, functional traits are useful parameters for studying plant hydric stress strategies and acclimating organ-level changes in plants when they are hydric-stressed, such as during drought or high precipitation (Rodríguez‐Ramírez et al., 2024a). We hypothesized that TMCF tree species display different wood and leaf anatomical adjustments or strategies in response to the air and soil moisture (Guerin et al., 2012; Thybring and Fredriksson, 2021). Similarly, plant trait information can be incorporated into functional trait theory and used to link plant performance to a range of specific climatic events in TMCF tree species (Báez et al., 2022). This has led to several predictions regarding how climatic variation in habitat drives specific functional and hydraulic architectural traits (Weigelt et al., 2021). To help resolve these questions, the main aims of this field study were to: (i) assess wood and leaf trait variability in ten TMCF tree species *in situ*; (ii) identify quantitative patterns (trait-trait and trait-climate relationships) among tree species; iii) evaluate how traits are influenced by climatic factors; and (iv) explore how climate influences tree species functional traits. Concatenating climate variation and wood- and leaf-anatomy are essential for understanding the growth, development, and adaptation of TMCF tree species under hydric stress (Pandey, 2021; Andrés-Hernández et al., 2023; Rodríguez‐Ramírez et al., 2024b).

## 2 Materials and methods

### 2.1 Study site

**Figure 1:**
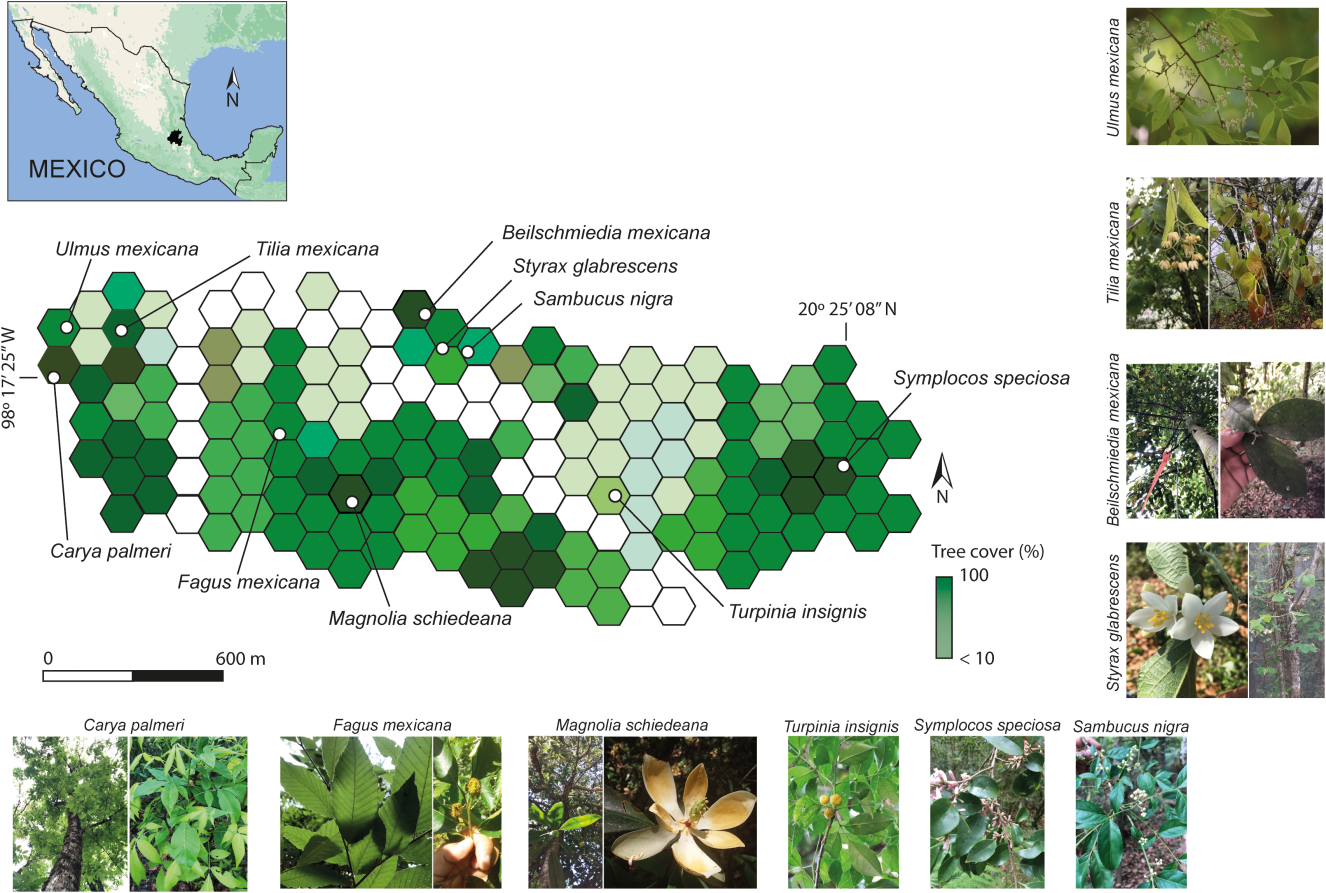
Map of the study area in the Medio Monte Natural Protected Area with hexagonal grids. The color scale represents the proportion of forest canopy cover in each grid (white: low canopy cover; dark green: high canopy cover). The location of the tree species is indicated by dots, and the inset map shows the study area within Mexico (black). Data acquired from https://globalforestwatch.org/map. The Tropical Montane Cloud Forest tree species assessed are represented.

The study area (longitudes: 98° 15’W; latitude: 20° 24’ N) is the Medio Monte Natural Protected Area (151.63 km^2^), altitudes ranging from 1558 to 1913 meters above sea level, located in central-eastern Sierra Madre Oriental, Mexico (**Figure 1**). The study forest exhibits an annual temperature range of 14.5 to 24.4°C and an average annual rainfall of approximately 2600 mm, with the majority occurring between late July and late October (Argüelles-Marrón et al., 2023). The study forest has acidic soils (pH 4–6), specifically andosol-humic and light sandy-loamy (Andrés-Hernández et al., 2023). The canopy (heights ranging from 4 to 40 m) comprises a mixture of Neotropical angiosperms (*Quercus* spp. *Fagus mexicana* Martínez, *Magnolia schiedeana* Schltdl., *Alnus jorullensis* Kunth, *Sambucus nigra* L., *Symplocos* spp., *Styrax glabrescens* Benth, *Tilia mexicana* Schltdl., *Turpinia insignis* (Kunth) Tul, *Beilchsmiedia* spp., *Clethra* spp., *Liquidambar styraciflua* L., *Carya* spp., and rarely *Ulmus mexicana* (Liebm.) Planch) and gymnosperms (*Pinus patula* Schltdl. & Cham.), among other species (Miranda and Sharp, 1950).

We used mean maximum and minimum temperature (T_MAX_, T_MIN_) in °C, vapor pressure deficit (V_PD_), monthly precipitation (P_RE_), and evapotranspiration (E_VT_) in mm from the Climatologies at High resolution for the Earth’s Land Surface Areas database (CHELSA v.2.0; http://chelsa-climate.org/; Karger et al., 2021). The layer resolution was approximately 1 km^2^ with records from 1980 to 2018. These climatic factors represent key aspects of the TMCF tree growth-climate relationship (Rodriguez-Ramirez et al., 2020a; Argüelles-Marrón et al., 2023).

### 2.2 Study species

For our study, we sampled ten individuals of each TMCF tree species: *Symplocos* sp*eciosa* Hemsl., *Sambucus nigra* L, *Styrax glabrescens*, *Turpinia insignis*, *Magnolia schiedeana*, *Tilia mexicana*, *Fagus mexicana*, *Beilschmiedia mexicana* (Mez) Kosterm., *Ulmus mexicana*, and *Carya palmeri* W.E. Manning. These species show a high relative abundance within the study area, so we chose them because they are typical of eastern Mexican TMCFs (Luna-Vega et al., 2022).

### 2.3 Sampling and processing

#### 2.3.1 Wood anatomy

From late December 2022 to early February 2023, exhaustive surveys were conducted to determine the distribution from the most abundant TMCF tree species in the study area (Kindt and Coe, 2005). We selected four individuals of each selected (excluding those exhibiting scars or rot) using a global positioning system (GPS) (Garmin^®^ 101 eTrex 10; Garmin, Olathe, KS, USA). Prior to the beginning of the rainy and growing seasons, we collected four wood samples from trees from each species (Rodríguez-Ramírez et al., 2018). Wood samples were collected by cutting from three to five cm wide piece containing bark and earlywood using a handsaw (Silky Zubat Handsaw, Truper^®^, CDMX, Mexico), and immediately fixed it in a formalin, acetic acid, and ethanol (FAA; 10:5:85) for 24 h. Histological sections were made in the transverse, longitudinal and radial planes at 25 μm width with the rotary microtome (Leica^®^ 2000R, Wetzlar, Germany). Samples were dehydrated with graded ethyl alcohol from 50% to 96% (v/v). Wood samples were stained with safranin dissolved in 96% alcohol for one hour and fast-green for eight seconds, then washed with 100% ethanol and rinsed with xylol (McCracken and Johansen, 1940). Finally, the samples were mounted on a synthetic resin (Ruzin, 2000). To gather data on vessel elements and fiber lengths, samples were macerated in Jeffrey’s solution as previously described (Berlyn and Miksche, 1976).

Eight anatomical wood traits were measured for each individual. Fifty measurements were taken per individual for each trait related to water transport (xylem vessel density, hydraulic diameter, vulnerability index, vessel grouping index, solitary vessel index, fiber length, length of uniseriate rays, and width of rays; **Table 1**; **Supplementary Figure S1A**), related to the moisture content of wood, and these relationships can vary among different tree species and under microclimatic conditions (Scholz et al., 2014; Schuldt et al., 2013; von Arx et al., 2013). The measurements were performed according to IAWA recommendations (IAWA COMMITTEE, 1989).

**Table 1:** Overview of wood and leaf traits, their acronyms, and measurements.

	Traits	Acronym	Measurements
Wood anatomical traits	Vessel density	V_D_	Vessel density= number of vessels per mm^2^
	Hydraulic diameter	D_H_	$D_{H}=\frac{\sum \;D^{5}}{\sum \;D^{4}}$
	Vulnerability index	V_I_	$VI=\;\frac{D}{V_{D}}$
	Vessel grouping	V_G_	$V_{G}=\;\frac{N_{vessels}}{N_{grouping}}$
	Solitary vessel index	V_S_	$V_{S}=\;\frac{N_{solitary\;vessels}}{N_{grouping}}$
	Fiber length	F_l_	The average length of fiber (μm)
	Length of uniseriate rays	L_UR_	The average length of uniseriate rays (μm)
	Width of rays	W_RY_	The average width of rays (μm)
Leaf morphological traits	Shape	*S*h	(1) Elliptic (2) Obovate (3) Ovate (4) Oblong (5) Linear (6) Special
	Leaf arrangement	*L*a	(3) Alternate (4) Subopposite (5) Opposite (6) Whorled
	Leaf organization	*L*o	(1) Simple (2) Compound (3) Palmately compound (4) Pinnately compound (5) Once compound (6) Twice, or bipinnately compound (7) Thrice compound
	Lamina length	*L*l	L= lm + la + lb
	Area of leaf size classes	*A*l	Leptophyll (<25 mm^2^), Nanophyll (25–225 mm^2^), Microphyll (225–2023 mm^2^), Notophyll (2025–4500 mm^2^), Mesophyll (4500–18225 mm^2^), Macrophyll (18225–164025 mm^2^), Megaphyll (164025 mm^2^).
	Apex angle	*A*a	Acute (< 90°), Obtuse (from 90° to 180°), Reflex (> 180°).
	Apex shape	*A*s	(1) Straight (2) Convex (3) Rounded (4) Truncate (5) Acuminate (6) Emarginate (7) Lobed
	Base angle	*B*a	Acute (< 90°), Obtuse (> 90° but< 360°), Reflex (> 180° but< 360°), Circular (> 360°).
	Number of basal veins	*N*b	Qualitative
	Vein density	*V*d	Sum of the length of all its segments (mm) per unit area (mm^2^).
	Agrophic veins	*A*v	(0) Present (1) Ausent
	Number of order of teeth	*N*t	One (all teeth are the same size or vary in size continuously), two (teeth are of two distinct sizes), three (teeth are of three distinct sizes).

#### 2.3.2 Leaf anatomy

We randomly selected 20 fully mature, healthy and undamaged leaves for each previously selected trees species (see Wood Anatomy section) in the summers (from May to June) of 2023 and 2024 (i.e. when the leaves reached their maximum development; Borchert et al., 2005). The leaves were selected from the basal branches of each tree, to minimize abiotic variation due to their position on the tree, atmospheric humidity levels, and light incidence. After collection, the leaves were placed in plastic bags (40 x 40 cm) with moist paper towels according to the method proposed by Rodríguez-Ramírez et al. (2021a). Leaf samples were transported to the Wood Anatomy Laboratory (Benemérita Universidad Autónoma de Puebla, BUAP, Mexico) within 3 days of collection.

The collected leaves were cleared in a 50% solution (w/v) of Na_2_CO_3_ at 85°C for 1 to 2 h. When leaf samples turned bright green, they were removed and rinsed with tap water. The leaves were then washed with a bleach solution 50% (Clorox^®^) for 45 to 50 min. To remove the epidermis, the leaves were placed in a glass container with bleach solution and tap water (50:50), and then brushed off with a soft marten hairbrush until the samples completely lost their greenness (Rodríguez-Ramírez et al., 2021b).

We digitized the 20 cleared leaves collected from each species. Each leaf was placed directly on the glass of a high-resolution flatbed scanner (HP OfficeJet Pro 7740, Hewlett-Packard Development Company, L.P., Houston, TX, USA). This method allowed us to obtain high-resolution digital images (1.3 µm per pixel resolution) with uniform illumination and minimal sample defects, as the resolution was high enough to zoom in to the finest veins.

We measured twelve data sets of leaf morphological traits: shape, leaf arrangement, leaf organization, lamina length, area of leaf size classes, apex angle, apex shape, base angle, number of basal veins, vein density, agrophic veins, and number of orders of teeth (**Table 1**; **Supplementary Figure S1B**) influencing water transport, light capture, and defense mechanisms (Ellis et al., 2009; Eller et al., 2016). Traits were measured at the mid‐leaf, taking care to avoid secondary veins. Leaf measurements (accuracy 0.01 mm) were performed using image analysis software (ImagePro v 4.5, Media Cybernetics, Carlsbad, CA) (Hickey et al., 1999; Guerin et al., 2012).

The assessment of differences between traits among species was carried out through one-way analysis of variance (ANOVA) and *post-hoc* Tukey’s test. Before analysis, the data were logarithmically transformed to improve normality and homoscedasticity. These analyses were performed using the R v.4.0.2 (R Core Team, 2018) and ggplot2 package (Wickham, 2016).

### 2.4 Data processing and analysis

#### 2.4.1 Links between functional traits and climatic factors

To assess the relationship between functional traits and climatic factors (T_MAX_, T_MIN_, V_PD_, P_RE_, and E_VT_) model-based fourth-corner analysis was employed. This approach addresses the “fourth-corner problem” by examining the connections among three matrices: (1) species by geographic coordinates, (2) species by functional traits, and (3) geographic coordinates by climatic factors, ultimately estimating a matrix that reflects trait-climate concatenations (Borcard et al., 2011). We adhered to the framework outlined by Warton et al. (2015), utilizing the R package mvabund (Wang et al., 2012). This process involved fitting a generalized linear model (GLM) where climatic variables were treated as functional traits, including their interactions. To enhance the accuracy of likelihood estimates, the model was fitted using a quasi-Poisson distribution for model errors, incorporating a LASSO-penalized regression model with the mvabund package (Wang et al., 2012). The model’s performance was then evaluated using diagnostic plots. All analyses were conducted using the *fourthcorner()* functions available in the R package ade4 (Dray et al., 2007).

#### 2.4.2 Multivariate analysis

We performed principal coordinate analysis (PCoA; Wang et al., 2022a) to identify dimensions of trait variability that maximally correlate with climatic factors (T_MAX_, T_MIN_, V_PD_, P_RE_, and E_VT_). In both analyses, presence/absence (1 or 0 respectively) scores were assigned to each trait category including traits which were originally represented by multiple categories (e.g., leaf size and shape). To assess climatic factors (distances) or similarities between functional traits to each TMCF tree species, we used the PCoA’s on the square root of the Bray–Curtis dissimilarities to obtain a fully Euclidean solution. All PCoA axes with eigenvalues >1 were retained, in this case the first four axes. When the distances are based on a complement-to-1 of a non-metric similarity coefficient, we eliminated the presence of negative eigenvalues using square-root transformation which makes it fully metric (Borcard et al., 2011). We extracted the first two axes of the analysis (Axis 1 and Axis 2) and used them in further analyses. The multivariate analyses were performed with CANOCO software v.5.0 (Šmilauer and Lepš, 2014).

#### 2.4.3 Cluster analysis

To explore whether certain trait syndromes (recurrent combinations of multiple traits that have evolved together within or across species; Raffard et al., 2017), we used k-means cluster analysis (Hartigan and Wong, 1979). Consistent occurrence could indicate that there are traits adapted to specific climatic factors, or multiple ecological strategies (e.g., acclimations at the leaf, stem, and root levels) to given environmental conditions. Nonetheless, k-means clustering operates in a Euclidean space by first using the absolute values (Legendre and Legendre, 1998), and is inversely related to simple concordance, which demonstrates an unwanted property that rare traits are treated as similar simply because they have a lot of zeros in common (Šmilauer and Lepš, 2014). Therefore, we created a modified Euclidean space by first, we used the absolute values of the Pearson’s correlation coefficients to perform a cluster analysis between traits, then performing a principal coordinate analysis (Zuur et al., 2007) on these values, before running the clustering in the space generated by the first two principal coordinate axes. We performed the analysis using hmisc (Harrell, 2024) and pheatmap (Kolde, 2022) packages. Based on this assessment, the traits were assigned to a consensus cluster, representing the cluster to which they were most frequently assigned. These clusters were superimposed on the PCoA plots to examine their position in climate space (De Bello et al., 2021).

#### 2.4.4 Climate influence on traits

To estimate smooth functional relationships between climate effects on cluster traits among TMCF tree species, we performed a generalized additive model (GAM; Wood, 2017) using a Poisson distribution. The explanatory factors were climatic factors (T_MAX_, T_MIN_, V_PD_, P_RE_, and E_VT_), whereas cluster traits were response variables (wood and leaf functional traits, **Table 1**; **Supplementary Figure S1**). To test the normality of the residuals of the response variables, we employed a Wilcoxon-Mann-Whitney test as outlined by Dehaene et al. (2021). A penalized smooth term prevents excess wiggliness and indicates the extent to which the fitted smooth function can vary. A wigglier function can capture intricate patterns in the data, while a less wiggly function results in a smoother more generalized fit (Wood, 2023). All the mixed models from this section can be compared using Akaike information criteria (AIC; Anderson et al., 1994) to determine the best fit model. Likewise, we use the effective degrees of freedom (edf), which at a maximum is the number of coefficients to be estimated in the model, minus any constraints (Pedersen et al., 2019). We performed all GAM analyses with R-software (R Core Team, 2018) using the glm2 (Donoghoe, 2022), lm4 (Bates et al., 2015), marginaleffects (Scholbeck et al., 2024), mumin (Barton, 2022) and ggplot2 (Wickham, 2016) packages.

## 3 Results

### 3.1 Trait behavior among TMCF tree species

**Figure 2:**
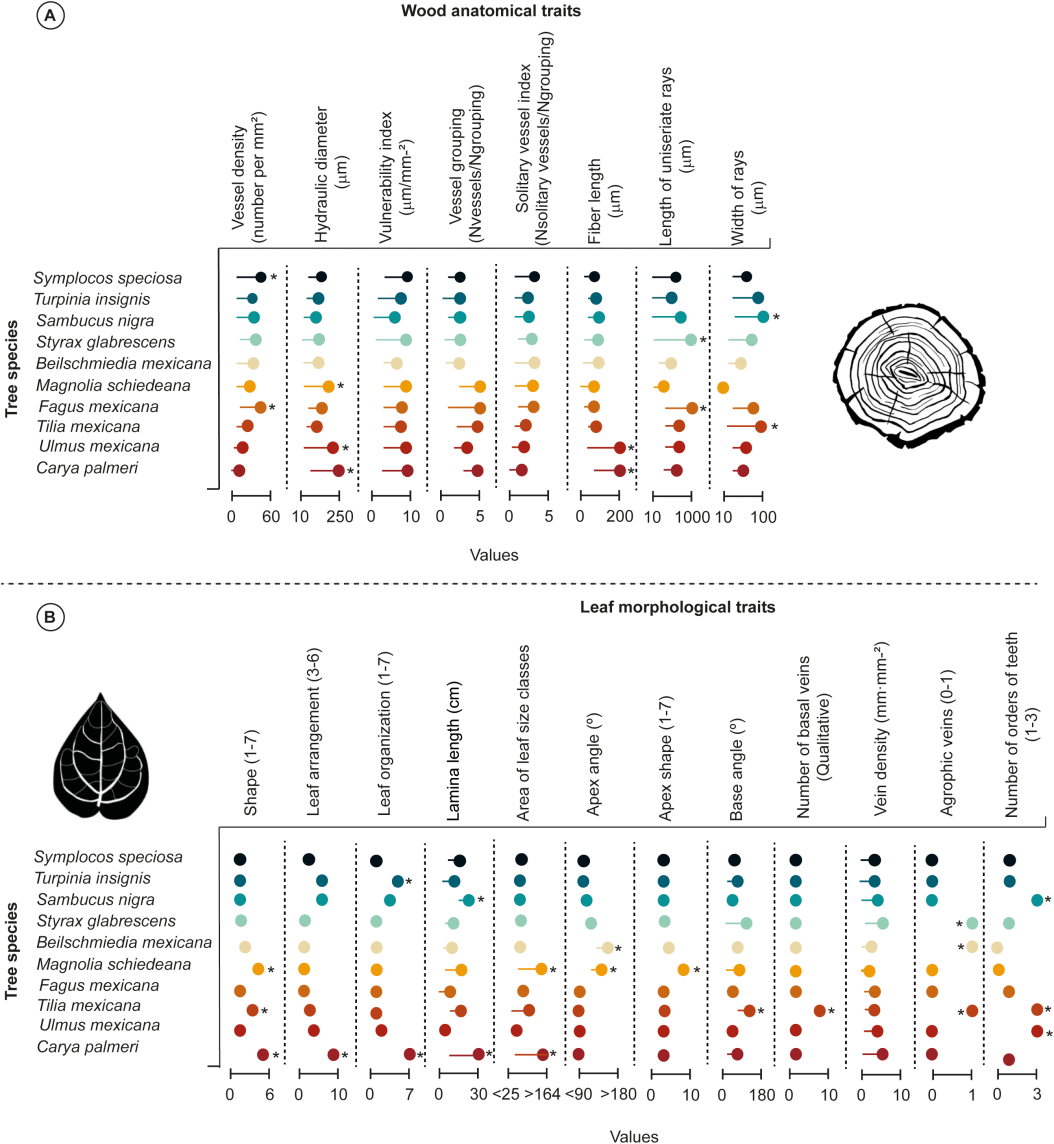
Lollipop plot illustrates all wood anatomical **(A)** and leaf morphological **(B)** traits variations among Tropical Montane Cloud Forest tree species. Lollipop plots with asterisks are significantly different as tested using a *post-hoc* Tukey test (*p*< 0.05). The asterisk denotes statistically significant differences between group means (*p*< 0.05).

We found high variations in wood anatomical traits among species (**Figure 2A**). Noteworthy, high vessel density (V_D_) values from 10 to 60 vessels per mm^2^) were identified in *S.* sp*eciosa* and *F. mexicana*; while *T. mexicana*, *U. mexicana*, and *C. palmeri* showed low vessels per mm^2^ (from 5 to 20). Likewise, we detected high hydraulic diameter (D_H_) values from 10 to 250 μm (i.e., *M. schiedeana*, *U. mexicana*, and *C. palmeri*), whereas narrow D_H_ values from 10 to 20 μm were detected in *T. insignis*, *S. nigra*, *S. glabrescens, B. mexicana*, *F. mexicana*, and *T. mexicana*). We observed a similar effect of variation among species in *M*. *schiedeana*, *F*. *mexicana*, *U*. *mexicana*, and *C. palmeri*, where exhibited high vulnerability index (V_I_) (from 5 to 10 μm/mm^-2^), and vessel grouping (V_G_) values (from 2 to 5 N_vessels_/N_grouping_); whereas *S.* sp*eciosa*, *T. insignis*, *S. nigra*, *S. glabrescens*, and *B. mexicana* exhibited constrained V_I_ (from 2 to 5), and V_G_ values (from 2 to 6). Notably, solitary vessel index (V_S_) values were similar among species (from 2 to 4 N_solitary vessels_/N_grouping_).

Regarding the vessel elements, specific deciduous tree species (i.e., *U. mexicana* and *C. palmeri*) showed high fiber length (F_L_) values from 5 to 200 μm compared to the other TMCF tree species, whereas in *S.* sp*eciosa, T. insignis, S. nigra, B. mexicana, M. schiedeana, F. mexicana*, and *T. mexicana* displayed narrow F_L_ values (from 2 to 30 μm). The length of uniseriate rays (L_UR_) was similar between *S. glabrescens* and *F. mexicana* (from 10 to 1000 μm), while *M. schiedeana* and *B. mexicana* showed the narrower L_UR_ values (L_UR_; from 10 to 20 μm). Finally, higher ray width (W_RY_) values were detected in *S. nigra* and *T. mexicana* (W_RY;_ from 10 to 100 μm), whereas *M. schiedeana* possessed narrower W_RY_ values (10 μm) (**Figure 2A**).

Our analysis demonstrated differences in leaf morphological traits among tree species using one-way ANOVA and *post-hoc* Tukey tests, that can strongly influence the ability of the species to adapt to fluctuations in the humidity of the environment (**Figure 2B**). Remarkably, *M*. *schiedeana* (obovate), *T*. *mexicana* (ovate), and *C*. *palmeri* (odd-pinnate) showed high plasticity in leaf shape (*S*h; from 5 to 6) compared to the other TMCF tree species surveyed. Leaf arrangement (*L*a) values were considerably narrower among tree species (from 3 to 5); notwithstanding, *C*. *palmeri* exhibited high *L*a values (from 5 to 6). Furthermore, *T. insignis* and *C*. *palmeri* showed high leaf organization (*L*o; from 6 to 7), where the values ranged from 1 to 4. Noteworthy, *M. schiedeana* and *C. palmeri* demonstrated high variation in lamina length (*L*l; from 15 to 30 cm) and area of leaf size classes (*A*l; ranged from > 30 to > 160; nanophyll) in relation to other species. Similar significant differences in apex angle (*A*a; ranging from 7 to 10°) and apex shape (*A*s; ranging from 8 to 10) were observed in *B. mexicana* and *M. schiedeana*. Likewise, *T. mexicana* displayed differences in base angle (*B*a; ≈170°), and number of basal veins (*N*b; ≈9) regarding to other species. Furthermore, *S. glabrescens*, *B. mexicana*, and *T. mexicana* showed high agrophic vein values (*A*v; 1); notwithstanding, *S. nigra*, *T. mexicana* and *U. mexicana* demonstrated high number of orders of teeth (*N*t; 3) (**Figure 2B**).

### 3.2 Relationship between traits and climatic factors

**Figure 3:**
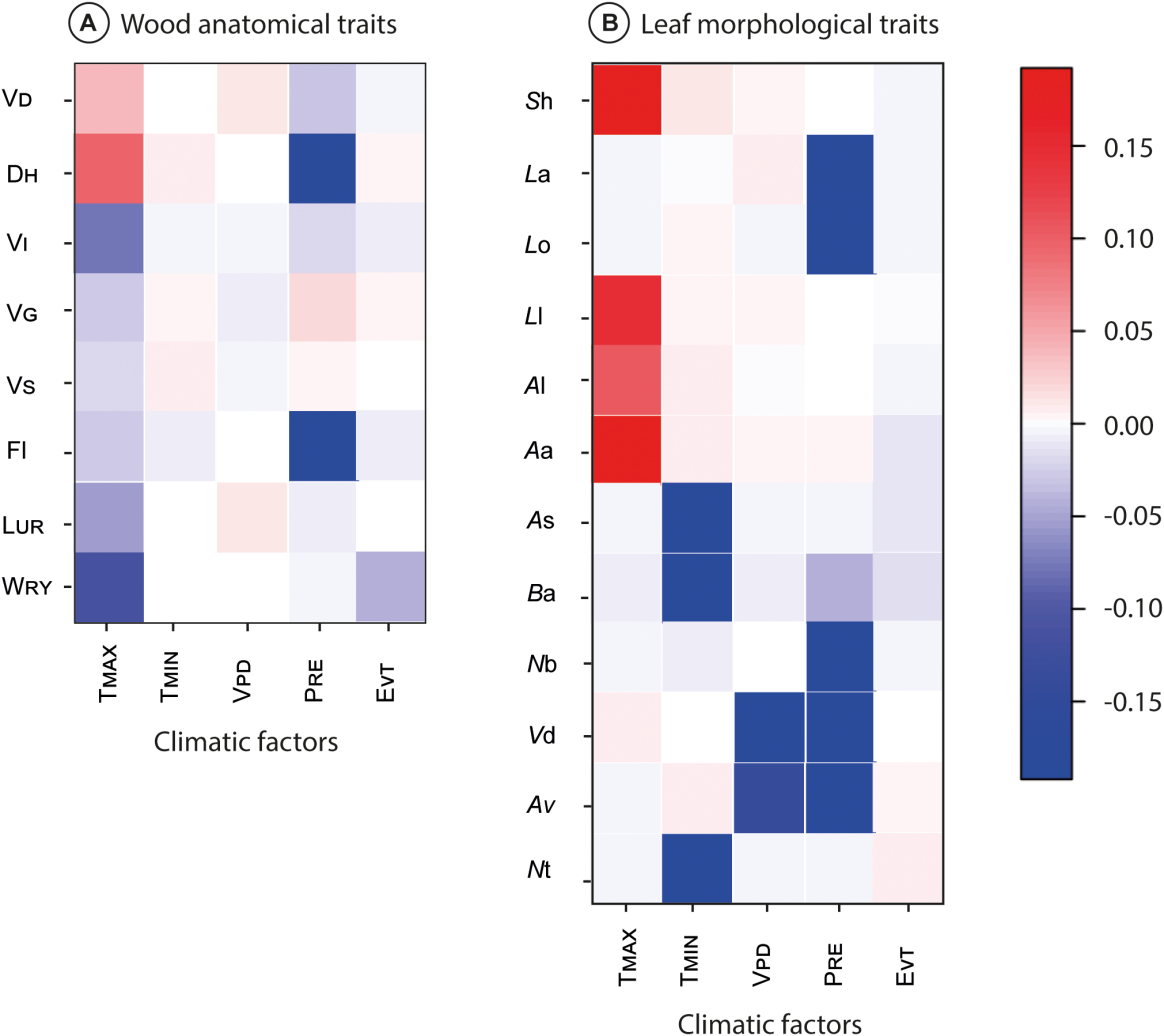
Results of the fourth‐corner analysis. Significant correlations between traits and climatic variables are represented by colored squares. Darker colors indicate stronger correlations. Wood anatomical traits: xylem vessel density (V_D_), hydraulic diameter (D_H_), vulnerability index (V_l_), vessel grouping index (V_G_), solitary vessel index (V_S_), fiber length (F_l_), length of uniseriate rays (L_UR_), and width of rays (W_RY_) **(A)**. Leaf morphological traits: leaf arrangement (*L*a), leaf organization (*L*o), lamina length (*L*l), area of leaf size classes (*A*l), apex angle (*A*a), apex shape (*A*s), base angle (*Ba*), number of basal veins (*N*b), vein density (*V*d), agrophic veins (*A*v), and number of orders of teeth (*N*t) **(B)**.

Overall, the fourth-corner analyses demonstrated significant association between trait-climatic factor interactions (**Figure 3**), 753 correlations significantly differed from zero, with an inertia of the overall model of 1345 (*p*< 0.001). Most TMCF tree species’ wood anatomical traits and leaf morphological traits and all climatic factors were significantly correlated with at least one other variable; V_D_ and D_H_ positively with T_MAX_ (from ≈0.05 to 0.15), whereas V_I_, V_G_. V_S_, F_l_, L_UR_ and W_RY_ exhibited negative correlation with T_MAX_ (from ≈-0.05 to -0.20; **Figure 3A**). The D_H_ and F_l_ showed a negative correlation with P_RE_ (-0.20; **Figure 3A**).

Of the leaf morphological traits, the *S*h, *L*l, *A*l, and *A*a demonstrated a positive correlation with T_MAX_ (from ≈0.10 to 0.20; **Figure 3B**); whereas *A*s, *B*a and *N*t showed negative correlation with T_MIN_ (-0.20), the *V*d and *A*v exhibited negative correlation with V_PD_ (from -0.10 to -0.15). Finally, the *L*a, *L*o, *N*b, *v*d, and *A*v showed negative correlation with P_RE_ (-0.20; **Figure 3B**).

### 3.3 Trait relationship

**Figure 4:**
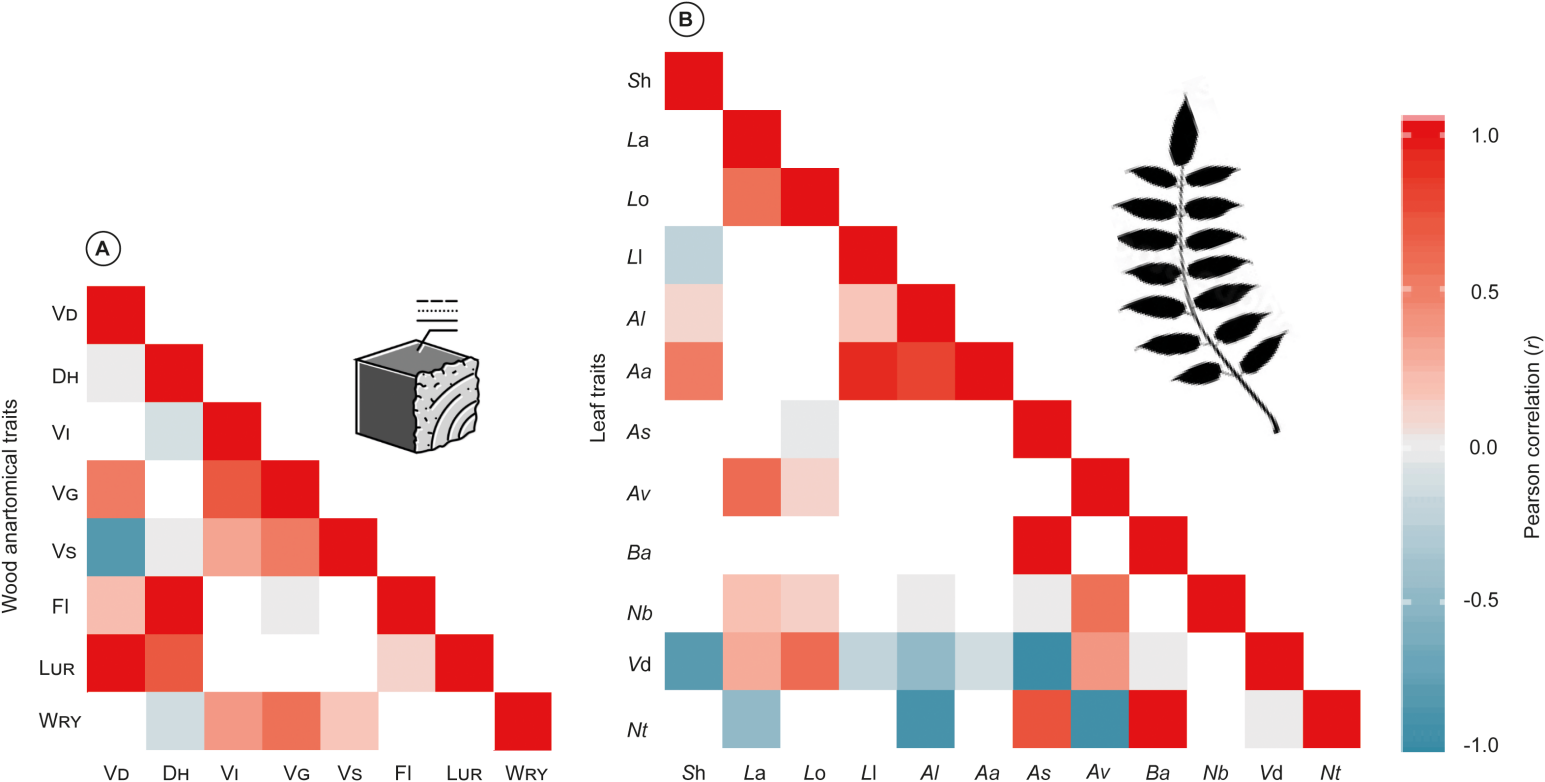
Pearson correlation (*r*) between wood anatomical **(A)**, and leaf morphological traits **(B)**. Red indicates the positive correlation, and blue demonstrates the negative correlation (*p*< 0.05 and *p*< 0.01, respectively). Trait abbreviations are given in **Table 1**.

The heatmap of the eight-wood anatomical and twelve-leaf morphological traits demonstrated different patterns and relationships between traits. Therefore, wood anatomical traits showed positive correlations between L_UR_ vs V_D_ and F_l_ vs D_H_, (*r*= 1.0), whereas a negative correlation between V_S_ vs V_D_ (**Figure 4A**). Moreover, regarding the leaf traits, the relation between *B*a vs *A*s and *N*t vs *B*a exhibited positive correlation (*r*= 1.0), and a noteworthy negative correlation between *V*d vs *S*h, *N*t vs *A*l, *V*d vs *A*s, and *N*t vs *A*v (*r*= -1.0) (**Figure 4B**).

### 3.4 Trait syndromes

**Figure 5:**
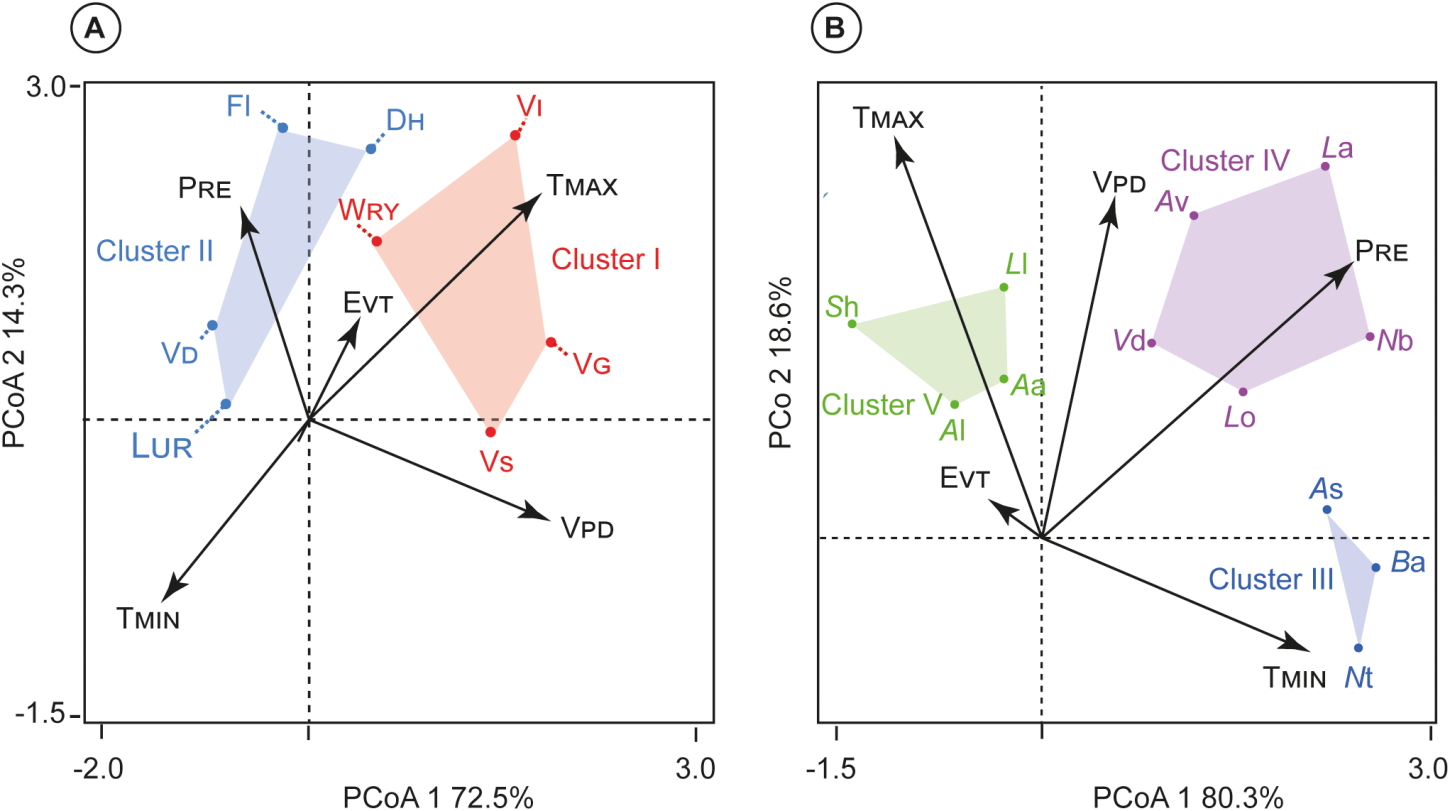
Clusters of traits from k-means cluster analysis. Traits in the same cluster are shown in space defined by climate-related trait dimensions from Principal Coordinate Analysis (PCoA). **(A)** Wood anatomical traits vs climatic factors; and **(B)** Leaf anatomical traits vs climatic factors. Trait abbreviations are given in **Table 1**.

Principal coordinate analysis (PCoA) revealed that traits were consistently grouped (i.e., Clusters I-V), and the location of these trait ‘syndromes’ in climate space was dissimilar between wood and leaf traits (**Figure 5**). They are a foundational concept for studying convergent evolution and the functional integration of traits in ecology and evolutionary biology. The PCoA did not suffer from collinearity among variables (for Pearson’s correlation coefficients) between the eight wood anatomical and twelve leaf traits surveyed (**Figure 4**). The trait syndromes were numbered according to their location on the dominant climate (Cluster I= V_S_, V_G_, W_RY_; Cluster II= L_UR_, V_D_ F_l_ and D_H_; Cluster III= *A*s, *B*a and *N*t; Cluster IV= *L*o, *V*d, *A*v, *L*a, and *N*b; and Cluster V= Al, Aa, *L*a, and *S*h).

Along the wood anatomical (Clusters I and II), axis 1 of PCoA was positively correlated with V_PD_ (*r*= 0.882) but negatively with T_MIN_ (*r*= −0.762). Axis 2 of PCoA was highly positively correlated with T_MAX_ (*r*= 0.843) and E_VT_ (*r*= 0.743) but negatively with P_RE_ (*r*= -0.876) (**Figure 5A**). The wood anatomical traits PCoA explained a total of 86.8% of the variability, with the first axis (72.5%) associated with T_MIN_ and V_PD_, whereas the second axis (14.3%) linked with P_RE_, and T_MAX_.

Along the leaf anatomical traits (Clusters III-V), axis 1 of PCoA was positively correlated with T_MIN_ (*r*= 0.896). Axis 2 of PCoA was highly correlated with P_RE_ (*r*= 0.978), V_PD_ (*r*= 0.651), but negatively correlated with T_MAX_ (*r*= -0.891), and E_VT_ (*r*= -0.631) (**Figure 5B**). Furthermore, the leaf traits PCoA described a total of 98.9% of the variability, the first axis (80.3%) related with T_MIN_; while the second axis (18.6%) associated with P_RE_, E_VT_, V_PD_, and T_MAX_.

### 3.5 Functional relationship between cluster-traits and climatic factors

**Figure 6:**
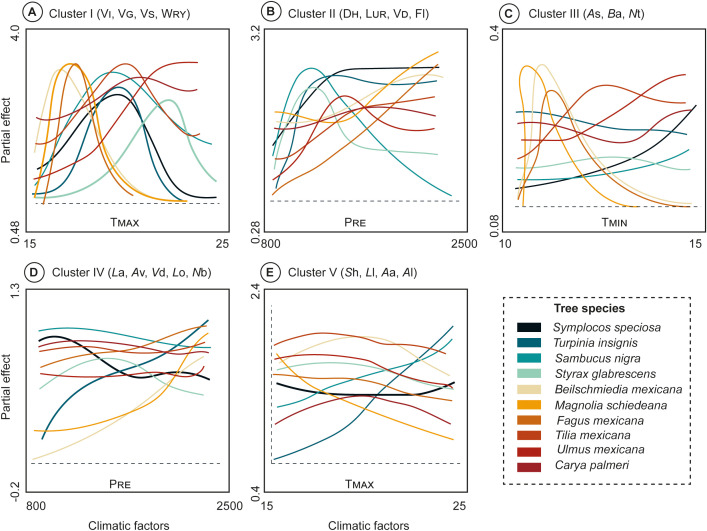
Response curves from the generalized additive models (GAM) according PCoA results of 10 TMCF tree species against specific climatic factors (T_MAX_, T_MIN_, P_RE_ and clusters. **(A)** Cluster I (V_I_, V_G_, V_S_, W_RY_) with T_MAX_; **(B)** Cluster II (D_H_, L_UR_, V_D_, Fl) with P_RE_; **(C)** Cluster III (*A*s, *B*a, *N*t) with T_MIN_; **(D)** Cluster IV (*L*a, *A*v, *V*d, *L*o, *N*b) with P_RE_; and **(E)** Cluster V (*S*h, *L*l, *A*a, *A*l) with T_MAX_. Trait abbreviations are given in **Table 1**.

The results of the GAM models (**Supplementary Table S1**) influenced the smooth function of the response on Clusters [wood anatomical (Cluster I-II), and leaf traits (Cluster III-V)] and the climatic relationship (**Figure 6**). The T_MAX_ effect on Cluster I (V_I_, V_G_, V_S_, W_RY_) demonstrated that the slope of *B. mexicana*, *M. schiedeana*, and *F. mexicana* exhibited similar shape, wiggliness and were positive up until T_MAX_ values approached 18°C. Similarly, *T. insignis*, *S. nigra*, *S.* sp*eciosa*, *T. mexicana*, and *C. palmeri* curves exhibited values ranged from 17 to 20°C, whereas *U. mexicana* and *S. glabrescens* showed wider values ranging from 21 to 23°C (**Figure 6A**). Likewise, the P_RE_ directly influenced on the Cluster II (D_H_, L_UR_, V_D_, and Fl) where *T. insignis*, *S. nigra* and *S*. *glabrescens* curves showed dissimilar shape and wiggliness with restricted values of 800 to 1000 mm, whereas *U. mexicana*, *S.* sp*eciosa* ranged from ≈1000 to 1300 mm. Noteworthy that, *B. mexicana*, *M. schiedeana, C. palmeri, T. mexicana*, and *F. mexicana* exhibit hydric efficiency with high precipitation values (> 2000 mm) (**Figure 6B**).

In addition, the T_min_ demonstrated a strong influence on the Cluster III (*A*s, *B*a, *N*t), where *M. schiedeana, B. mexicana*, and *F. mexicana* curves were constrained to values ranging from 11 to 13°C; even though *S.* sp*eciosa, T. insignis, S. nigra, S. glabrescens, T. mexicana, U. mexicana*, and *C. palmeri* curves showed wider values from 13 to 15°C (**Figure 6C**). In brief to assess the P_RE_ effect, the Cluster IV (*L*a, *A*v, *V*d, *L*o, *N*b), the *S.* sp*eciosa, T. insignis, S. nigra, S. glabrescens, F. mexicana*, *T. mexicana*, *U. mexicana*, and *C. palmeri* curves demonstrated a very similar overall shape but differ in their wiggliness, with values ranging from 800 to 2500 mm; whereas *B. mexicana* and *M. schiedeana* curves showed similar shape and wiggliness with values of ≈2500 mm (**Figure 6D**).

Finally, the T _max_ on Cluster V (*S*h, *L*l, *A*a, *A*l), we observed a similar shape among *F. mexicana*, *S. glabrescens*, *S.* sp*eciosa*, *B. mexicana*, *C. palmeri*, *T. mexicana*, and *U. mexicana* curves; notwithstanding different wiggliness with values ranging from 15 to 25 °C. Moreover, *T. insignis* and *S. nigra* demonstrated similar shape but differ in wiggliness with values fluctuating from 20 to 25 °C, whereas *M. schiedeana* curve exhibited negative effect (**Figure 6E**).

## 4 Discussion

### 4.1 Towards an understanding of wood and leaf anatomical traits

Although we noted similar environmental conditions across the TMCF study area, our research suggests that each tree species has developed unique adaptations that allow it to adjust to the region’s macroclimatic changes (Eller et al., 2020; Zahedi et al., 2024). Nonetheless, the wood and leaf traits identified in this research indicate a particular ability to acclimate. The presence of TMCF tree species, such as *F*. *mexicana*, *U*. *mexicana*, *C*. *palmeri*, and *M*. *schiedeana*, are sensitive to changes in climate, as noted by several authors (Vásquez-Morales et al., 2014; Rodríguez-Ramírez et al., 2018; Ames-Martínez et al., 2022), indicate that they possess specific morphological characteristics that may increase their adaptive or resilience responses to fog and/or mist immersion changes (Sánchez-Velásquez et al., 2023).

It is noteworthy that *M. schiedeana*, *U. mexicana*, and *C. palmeri* exhibited significantly higher hydraulic diameter values than the other species, suggesting that they may influence the resilience of TMCF tree species to drought by enhancing water transport capacity, reducing vulnerability to cavitation, facilitating adaptive plasticity, improving growth recovery rates, and supporting ecological interactions within these unique forest ecosystems (Rodríguez-Ramírez et al., 2020a, 2022; Rodríguez‐Ramírez et al., 2024a). High-vessel density enhances drought resilience through thicker cell walls but reduces growth rates by 30-50% compared to low-vessel density (Eller et al., 2020; Mo et al., 2024). The high-vessel density presence in particular TMCF tree species (*F. mexicana* and *S. speciosa*) contribute to habitat diversity by supporting different microhabitats (López-Calvillo et al., 2023) and shaping the functioning of TMCFs (Hu and Riveros-Iregui, 2016; Eller et al., 2020).

The presence of high fiber length in *U*. *mexicana*, and *C*. *palmeri* contribute to the formation of strong, flexible and cohesive wood tissue. This structural advantage allows trees to grow larger and taller, increasing the potential for above-ground biomass accumulation and consequently higher carbon storage in living trees (Bukoski et al., 2022). It also increases mechanical strength to prevent canopy collapse under hydric stress (Brum et al., 2023). This is particularly important during periods of hydric stress (Rodríguez‐Ramírez et al., 2024b), as trees may experience increased vulnerability to physical damage from wind or heavy rainfall. A robust fiber structure helps maintain stability, allowing trees to withstand climatic stresses (i.e., soil moisture availability) that often accompany drought conditions (DeBell et al., 2002; Yang et al., 2024). This stress affects traits in TMCF tree species, influencing growth, and anatomy (Eller et al., 2020; Rodríguez‐Ramírez et al., 2024b). The high varying uniseriate ray length (i.e., *S. glabrescens* and *F*. *mexicana*) and wide width of rays (*S. nigra* and *T*. *mexicana*) facilitate hydraulic pathways, and store non-structural carbohydrates and other nutrients (von Arx et al., 2015). This storage is vital for the TMCF tree’s energy management, particularly during specific phenological processes (i.e., masting events; Rodríguez-Ramírez et al., 2019) or environmental stress (i.e., drought events, freezing; Rodríguez-Ramírez et al., 2023a). Likewise, the wood mechanical properties of these rays help trees to survive in windy, rainy conditions (Ayala-Usma et al., 2019). This connectivity is crucial for maintaining physiological balance, especially in the humid and variable conditions of TMCFs, where moisture levels can daily fluctuate significantly (Borchert et al., 2005; Fahey et al., 2016). This acclimation is crucial in dense TMCFs (i.e., Mexican beech forests; Rodríguez-Ramírez et al., 2016), where light is in short supply.

Nevertheless, the distinctive leaf morphology suggests that the TMCF tree species evolved to thrive in the diverse moisture conditions. Even though these phenotypic traits could be coded by independent genes, and modified by different selective pressures (Sobral, 2021), the leaf anatomical traits are mechanistically related and will cohere (Wright et al., 2017; Wang et al., 2022a), and may have advantages in one moisture environment and costs in another (Givnish, 1984; Guerin et al., 2012; Sánchez-Velásquez et al., 2023). Many tree species in TMCFs exhibit structural characteristics of leaves that enhance their ability to utilize the condensation of water droplets from fog and/or mist, which can then be absorbed directly into the plant system (Royer et al., 2012; Oliveira et al., 2014; Hughes et al., 2024). This morphological variation within species along TMCF communities suggests adaptation, which may allow local persistence and migration of adaptive potential, or at least response to moisture variation (Ackerly et al., 2002; Hughes et al., 2024). The presence the *M*. *schiedeana*, *T*. *mexicana*, and *C*. *palmeri* displayed a larger size of leaf, area of leaf size classes compared with smaller leaf species (i.e., *F*. *mexicana*, *S*. *speciosa*, *U*. *mexicana, T. insignis*, and *B. mexicana*), maximizing surface area (i.e., nanophyll with intermediate precipitation seasonality; Givnish, 1984; Wang et al., 2022b) for absorbing moisture from the humid air (i.e., vapor plumes, fog, mist, and drizzle), maintaining hydration during drier periods when rainfall may be scarce (Liancourt et al., 2015; Wright et al., 2017; Wang et al., 2022b). Nonetheless, *M*. *schiedeana* and *B*. *mexicana* with apex angle (≥ 180°), and apex shape (i.e., convex, rounded, and acuminate) facilitate the runoff of water from fog or mist, ensuring that excess moisture does not accumulate on the leaf surface (Graham and Christopher, 2023). This adaptation prevents fungal infections and other moisture-related problems in the subcanopy of TMCFs (Cheek et al., 2023).

In particular, *T. mexicana* exhibited a notably wider leaf angle base (> 170°, asymmetrical leaf bases) than the other TMCF tree species, which allows for better light capture and photosynthesis (McCarthy and Mason-Gamer, 2020; Ramírez-Díaz et al., 2024), especially in Lower TMCFs where light conditions can be variable (Rodríguez-Ramírez et al., 2024a). Besides, the presence of agrophic veins in specific tree species (i.e., *S. glabrescens*, *B*. *mexicana*, and *T*. *mexicana*) enabling the passive diffusion of water, nutrients (e.g., sugar, hormone auxin), regulating cell-to-cell communication (Band, 2022). This characteristic has been reported in other species from moist environments, such as *Bernardia* species (Cervantes et al., 2009), and extinct species as *Tilia populifolia* H.T. Chang, *Corylopsis reedae* Radtke, Pigg et Wehr, *Fothergilla malloryi* Radtke, Pigg et Wehr, and *Apeibopsis atwoodii* Hollick (Radtke et al., 2005; Carvalho et al., 2011).

Finally, toothed leaves are more common in areas with high rainfall and lower temperatures, such as the TMCF, demonstrating a complex concatenation among morphology, physiology, and environmental adaptation. Similarly, toothed leaves in TMCF tree species are primarily associated with strategies that maximize early-season photosynthesis and rapid growth in cool, wet, and water-abundant environments, leveraging the unique climatic and hydrological conditions of TMCFs (Givnish, 1984; Royer and Wilf, 2006). *U. mexicana*, *T*. *mexicana* and *S*. *nigra* exhibited high number of the order teeth, leaf hydration and function during dry periods (Iszkulo et al., 2024); notwithstanding, this limits leaf longevity, offering probably an early season opportunity for rapid photosynthesis (Baker-Brosh and Peet, 1997). Notably, *M*. *schiedeana* displays leaves that are broadly oblong-elliptical in shape with untoothed margins (Rodríguez-Ramírez et al., 2020a), which may help to low leaf nitrogen concentration and reduce the likelihood of hydric stress during drought events (Royer et al., 2012). This is because loss of cellular turgor would not result in leaf wilting or collapse (Postek, 1981), involving the movement of xylem sap and the resultant carbon economy of the TMCF tree species (Royer and Wilf, 2006; Royer et al., 2012).

### 4.2 Climatic effect on functional trait syndromes

Several of the individual functional trait–climatic factor correlations that we observed support the functional trait theory (Kearney et al., 2021; Viliani et al., 2024), such as specific wood anatomical traits (D_H_ and V_D_) and leaf morphological traits (*S*h, *L*l, *A*l, and *A*a). According to Kühn et al. (2021), functional trait stability may influence performance or fitness in fluctuating climates. Although the observed correlations are descriptive, they are predictive of traits that are likely to change in response to environmental variation (Sánchez‐Velásquez et al., 2023). Correlations between functional traits and climatic factors provide a reference point for identifying environmental variables associated with traits exhibited by relict-endemic TMCF tree species (Rodríguez-Ramírez et al., 2023b; Rodríguez-Ramírez et al., 2024a).

The wood and leaf traits adjust among TMCF tree species because of the changing environmental conditions (Toledo-Aceves et al., 2019; Yang et al., 2021; Rodríguez-Ramírez et al., 2024b). T_MAX_ triggers a synergy among wood anatomical traits (Cluster I: V_S_, V_G_, W_RY_ and V_I_) is an adaptive trait that allows plants to optimize water transport according to their ecological niches (von Arx et al., 2013; Scholz et al., 2014). The combination of historical maximum temperature ranges with seasonal variations and anticipated future climate data for tropical tree species (Ponce-Reyes et al., 2012), could result in a reduction in trait-fitness in climate change (Vasseur et al., 2014; Wright and Francia, 2024). This is because of an increased likelihood of these species experiencing temperatures above their critical thermal tolerances, which may lead to slower growth (Guillemot et al., 2022; Feeley et al., 2023).

Considering the crucial role that cloud uplift events play in the TMCF water budget, a reduction in the frequency of fog, but rainier climate is likely to result in increased evapotranspiration, vegetation hydric stress and, subsequently, plant mortality (Oliveira et al., 2014; Zhang et al., 2024). For instance, TMCF species in less moist environments might have evolved narrower vessels to enhance safety (Anfodillo and Olson, 2021; Argüelles-Marrón et al., 2023), whereas those in wetter environments might allowing for adequate hydraulic connection in specific xylem vessel traits (i.e., L_UR_, D_H_, V_D_, and F_l_), preventing mortality through reduced resilience to hydric stress (Rodríguez‐Ramírez et al., 2024b).

Tropical trees subjected to hydric stress exhibit morpho-anatomical changes that are closely linked to cellular, physiological and biochemical acclimations aimed at minimizing water loss through transpiration and optimizing water use efficiency (Amesalu and Kebede, 2020; Eller et al., 2020; Zahedi et al., 2024). Significant concatenation among leaf morphological traits can indicate adaptation in response to shifts in climate (Givnish, 1984; Doughty et al., 2018; Manishimwe et al., 2022). It is evident that the identified clusters are subject to influence from climatic factors, including V_PD_, T_MAX_, P_RE_, and T_MIN_. These syndromes allow for maximum fitness under hydric stress, resulting in specific morphological adaptations to TMCF conditions (Andrés-Hernández et al., 2023; Rodríguez-Ramírez et al., 2023a; Rodríguez-Ramírez et al., 2023b). Due to the considerable diversity in hydraulic trait assemblages, it is currently impossible to determine the leaf hydraulic conductivity and vulnerability to hydric stress of TMCFs (Liancourt et al., 2015). Furthermore, the potential risk of hydraulic failure is also unknown (Mackay, 2024; Robbins et al., 2024), given the lack of available data on climatic factors such as the vapor pressure deficit (V_PD_), precipitation (P_RE_), maximum temperature (T_MAX_) and minimum temperature (T_MIN_). It is therefore possible that ecological niche conservatism has influenced the persistence of TMCF tree species, limiting the distribution of ecologically dissimilar functional traits across moist or temperature variation in specific regions (Saldaña-Acosta et al., 2008).

The adaptative interspecific capability differences in leaf anatomical traits suggest a wide diversification of the hydraulic strategy among TMCF tree species (Apgaua et al., 2022; Manishimwe et al., 2022). Nevertheless, our research demonstrates that the probability of leaf morphological variations during low moisture periods depend on an understanding of the interaction between leaf anatomical trait assemblages (Robbins et al., 2024). Conversely, the clustering of traits (i.e., *A*s, *B*a, *N*t, *L*a, *A*v, *V*d, *L*o, *N*b, *S*h, *L*l, *A*a, and *A*l) may demonstrate hydraulic adjustments that triggers a mechanical safety versus water transport efficiency tradeoff (Tng et al., 2018), indicating that leaf morphology and phenology reflect a set of ecological strategies that can co-vary with anatomical hydraulic traits (Apgaua et al., 2022). Additionally, the leaf morphological in some evergreen species (e.g., *M. schiedeana*, *S. speciosa*, *B. mexicana*) and semideciduous tree species (e.g., *F. mexicana*) (**Figure 2**) supports the argument that these species may have a hydraulic adjustment that makes them highly sensitive to hotter climates (Nishida and Christophel, 1999; Rodríguez-Ramírez et al., 2020b; Rodríguez-Ramírez et al., 2021a; Reyes-Ortiz et al., 2024).

Functional trait syndromes varied significantly among TMCF tree species, with unique trait combinations (Clusters) emerging for specific climatic responses. These results are in accordance with those previously reported by (Nishida and Christophel, 1999; Vogt, 2001; Rodríguez-Ramírez et al., 2018; Rodríguez-Ramírez et al., 2020b; Rodríguez-Ramírez et al., 2021b; Rodríguez-Ramírez et al., 2024a; Bartholomew et al., 2020; López-Calvillo et al., 2023; Aranda et al., 2024; Ramírez-Díaz et al., 2024; Reyes-Ortiz et al., 2024), where the TMCF species as *F*. *mexicana*, *T*. *mexicana*, *M*. *schiedeana*, *B*. *mexicana*, *S*. *nigra*, *S*. *speciosa*, and *C*. *palmeri* exhibited specific wood and/or leaf trait acclimation and/or adaptation strategies to moisture variation, suggesting divergent relationships between trait values and fitness in a particular context are defined by multiple selective coefficients (Sobral, 2021), that shape the evolutionary course of TMCF communities (Price et al., 2011). Further research is required to gain a deeper understanding of the direct influence of climate on the relict-endemic TMCF tree species in phenological processes (e.g., mass flowering, cribo-xylogenesis).

The resilience of TMCFs depends on balancing trait-mediated trade-offs between drought tolerance and productivity (Locatelli et al., 2015). While conservative traits buffer against moderate drought, their slow growth rates may hinder recovery from extreme disturbances (Saatchi et al., 2021). Conversely, acquisitive strategies risk hydraulic failure under prolonged drought but maintain faster carbon cycling (Peters et al., 2019). The interplay between fog persistence, temperature rise, and moisture variability will determine whether these ecosystems can functionally adapt or face compositional collapse. This will enable us to provide a better understanding of resilience, resistance and recovery to changes in moisture conditions, and identify a reliable and effective choice for future restoration and conservation strategies. Targeted conservation should prioritize areas of high functional diversity to maintain adaptive capacity (Goldsmith et al., 2017; Forzieri et al., 2022).

## 5 Conclusions

In this paper we demonstrated that some TMCF tree species exhibit different wood and leaf anatomical adjustments or strategies in response to the wet environment. This highlights the importance of wood and leaf anatomy trait study for TMCF tree species and indicates that climate variation may alter acclimation and adaptation capacity among species. Finally, our study provides a framework that could be used to begin using functional trait theory to understand the relative importance of different growth patterns, total photosynthetic rate, leaf chlorophyll content and transpiration on the ability of TMCF tree species to survive periods of hydric stress. Future work should test the observed patterns using bark, cribo-xylogenesis to three distinct types of TMCFs: Lower (LTMCF; >700–1700 m asl), Upper (UTMCF; 1701–1799 m asl), and Subalpine (STMCF; 1800–3500 m asl). These studies will improve the representation of plant hydraulics within the montane ecosystem and help to understand phenological processes such as growth, development, and acclimation of TMCF tree species during periods of hydric deficit and refine predictions of how future climate change will affect TMCF functional traits.

## Data Availability

The original contributions presented in the study are included in the article/**Supplementary Material**. Further inquiries can be directed to the corresponding author/s.
