# Supplementary material for: Does climatic variation drive the adjustment of functional traits? An assessment of Tropical Montane Cloud Forest tree species
Source: Front Plant Sci. 2025 Jun 4;16:1555607. doi: 10.3389/fpls.2025.1555607 (PMC12175852; doi:10.3389/fpls.2025.1555607)
Supplement: Supplementary file 1 [file DataSheet1.docx]

Supplementary Material

## Supplementary Figure 1


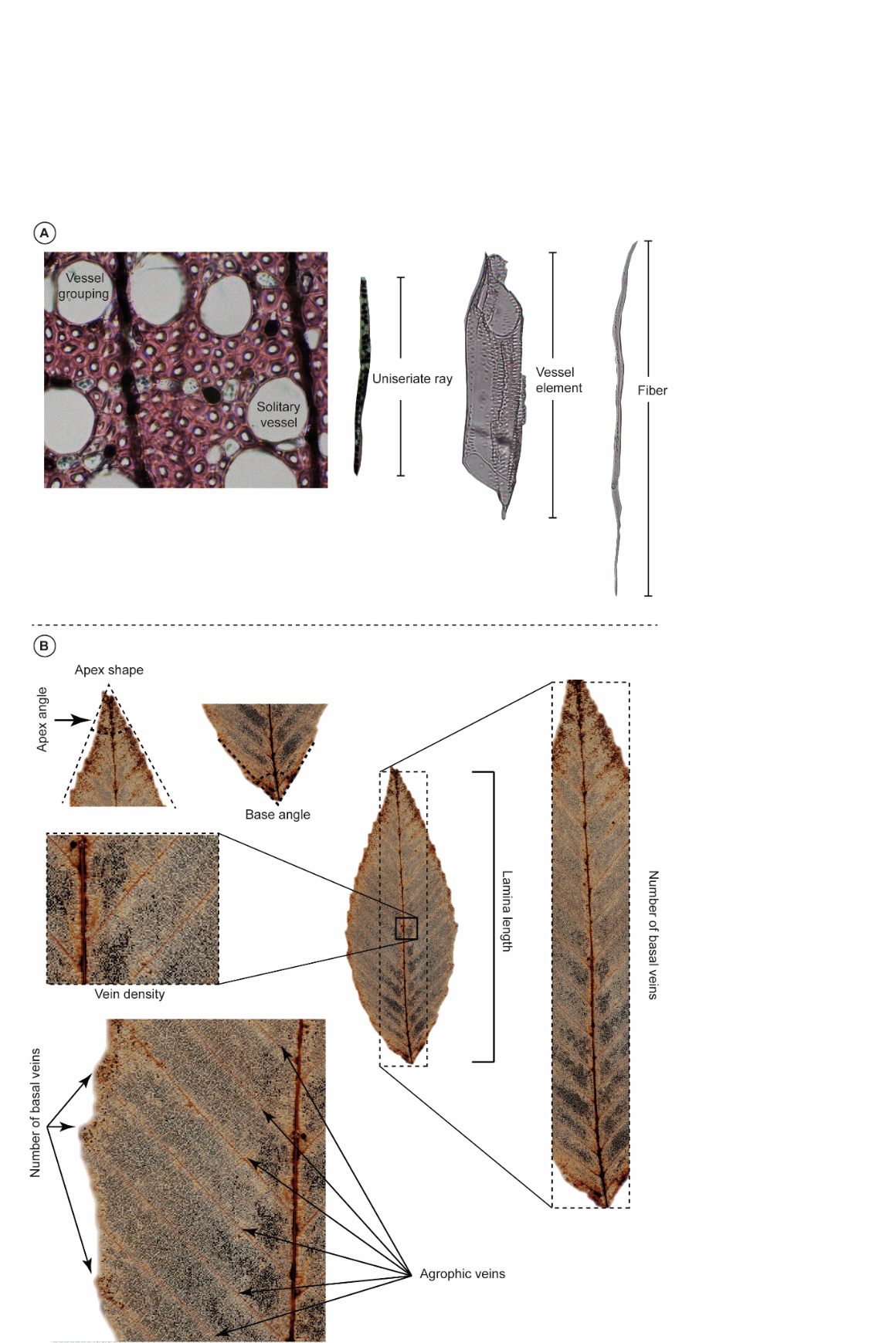


**Supplementary Figure 1.** Schematic representation of functional trait assessment. A) Wood anatomical traits. B) Leaf morphological trait measurements.

|  |  | **Tropical montane cloud forest tree species** | | | | | | | | | | | | | | | | | | | | | | | | | | | | | |
| --- | --- | --- | --- | --- | --- | --- | --- | --- | --- | --- | --- | --- | --- | --- | --- | --- | --- | --- | --- | --- | --- | --- | --- | --- | --- | --- | --- | --- | --- | --- | --- |
|  |  | ***Carya palmeri*** | | | ***Ulmus mexicana*** | | | ***Tilia mexicana*** | | | ***Fagus mexicana*** | | | ***Magnolia schiedeana*** | | | ***Beilchsmiedia mexicana*** | | | ***Styrax glabrescens*** | | | ***Sambucus nigra*** | | | ***Turpinia insignis*** | | | ***Symplocos speciosa*** | | |
| **Wood anatomical traits** | **Factor** | R^2^ | edf | *p* | R^2^ | edf | *p* | R^2^ | edf | *p* | R^2^ | edf | *p* | R^2^ | edf | *p* | R^2^ | edf | *p* | R^2^ | edf | *p* | R^2^ | edf | *p* | R^2^ | edf | *p* | R^2^ | edf | *p* |
| V_D_ | s(indiv) | 0.69 | 1.00 | * | 0.66 | 1.00 | * | 0.58 | 1.00 | ** | 0.70 | 1.00 | * | 0.69 | 1.00 | * | 0.58 | 23.0 | ** | 0.66 | 1.00 | ** | 0.65 | 1.00 | ** | 0.71 | 1.00 | * | 0.75 | 1.00 | * |
|  | s(T_MAX_) |  | 1.08 |  |  | 1.00 |  |  | 1.00 |  |  | 2.10 |  |  | 1.00 |  |  | 1.00 |  |  | 1.00 |  |  | 1.00 |  |  | 1.00 |  |  | 1.00 |  |
|  | s(T_MIN_) |  | 1.00 |  |  | 1.00 |  |  | 1.00 |  |  | 1.00 |  |  | 1.00 |  |  | 1.00 |  |  | 1.00 |  |  | 1.00 |  |  | 1.00 |  |  | 1.00 |  |
|  | s(V_PD_) |  | 1.00 |  |  | 1.00 |  |  | 1.00 |  |  | 1.00 |  |  | 1.00 |  |  | 1.00 |  |  | 1.00 |  |  | 1.00 |  |  | 1.00 |  |  | 1.00 |  |
|  | s(P_RE_) |  | 1.00 |  |  | 1.00 |  |  | 26.0 |  |  | 28.0 |  |  | 30.0 |  |  | 1.00 |  |  | 1.00 |  |  | 1.00 |  |  | 1.00 |  |  | 1.00 |  |
|  | s(E_V_T) |  | 1.10 |  |  | 1.00 |  |  | 1.00 |  |  | 1.00 |  |  | 1.00 |  |  | 1.00 |  |  | 1.10 |  |  | 1.00 |  |  | 1.00 |  |  | 1.00 |  |
|  | s(period) |  | 0.88 |  |  | 0.87 |  |  | 0.91 |  |  | 0.94 |  |  | 0.90 |  |  | 0.86 |  |  | 0.78 |  |  | 0.70 |  |  | 0.77 |  |  | 0.82 |  |
|  |  |  |  |  |  |  |  |  |  |  |  |  |  |  |  |  |  |  |  |  |  |  |  |  |  |  |  |  |  |  |  |
| **D_H_** | s(indiv) | 0.56 | 1.00 | ** | 0.76 | 1.00 | * | 0.66 | 1.00 | ** | 0.61 | 1.00 | ** | 0.43 | 1.00 | *** | 0.72 | 1.00 | * | 0.76 | 1.00 | * | 0.74 | 1.00 | * | 0.63 | 1.00 | ** | 0.61 | 1.00 | *** |
|  | s(T_MAX_) |  | 1.00 |  |  | 3.00 |  |  | 2.00 |  |  | 2.10 |  |  | 1.11 |  |  | 1.10 |  |  | 1.00 |  |  | 1.00 |  |  | 1.00 |  |  | 1.00 |  |
|  | s(T_MIN_) |  | 1.00 |  |  | 1.00 |  |  | 1.00 |  |  | 1.00 |  |  | 1.00 |  |  | 1.00 |  |  | 1.00 |  |  | 21.0 |  |  | 1.00 |  |  | 1.00 |  |
|  | s(V_PD_) |  | 1.00 |  |  | 1.00 |  |  | 1.00 |  |  | 1.00 |  |  | 1.00 |  |  | 1.00 |  |  | 1.00 |  |  | 1.00 |  |  | 1.00 |  |  | 1.00 |  |
|  | s(P_RE_) |  | 1.00 |  |  | 1.90 |  |  | 1.90 |  |  | 28.0 |  |  | 18.0 |  |  | 1.00 |  |  | 1.00 |  |  | 1.00 |  |  | 1.00 |  |  | 1.00 |  |
|  | s(E_V_T) |  | 1.00 |  |  | 1.00 |  |  | 1.00 |  |  | 1.00 |  |  | 1.00 |  |  | 1.00 |  |  | 1.10 |  |  | 18.0 |  |  | 1.00 |  |  | 1.00 |  |
|  | s(period) |  | 0.89 |  |  | 0.76 |  |  | 0.85 |  |  | 0.94 |  |  | 0.70 |  |  | 0.88 |  |  | 0.78 |  |  | 0.61 |  |  | 0.65 |  |  | 0.72 |  |
|  |  |  |  |  |  |  |  |  |  |  |  |  |  |  |  |  |  |  |  |  |  |  |  |  |  |  |  |  |  |  |  |
| **V_I_** | s(indiv) | 0.60 | 1.00 | ** | 0.71 | 1.00 | * | 0.77 | 1.00 | * | 0.76 | 1.00 | * | 0.52 | 1.00 | *** | 0.87 | 1.00 | * | 0.54 | 1.00 | *** | 0.77 | 1.00 | * | 0.87 | 1.00 | * | 0.73 | 1.00 | * |
|  | s(T_MAX_) |  | 1.00 |  |  | 3.00 |  |  | 1.11 |  |  | 1.00 |  |  | 30.1 |  |  | 1.00 |  |  | 1.00 |  |  | 1.00 |  |  | 1.00 |  |  | 1.00 |  |
|  | s(T_MIN_) |  | 1.00 |  |  | 1.00 |  |  | 1.00 |  |  | 1.00 |  |  | 1.00 |  |  | 1.00 |  |  | 1.00 |  |  | 23.0 |  |  | 1.00 |  |  | 1.00 |  |
|  | s(V_PD_) |  | 1.00 |  |  | 1.00 |  |  | 1.00 |  |  | 1.05 |  |  | 1.10 |  |  | 11.00 |  |  | 1.00 |  |  | 1.00 |  |  | 1.00 |  |  | 1.00 |  |
|  | s(P_RE_) |  | 1.00 |  |  | 1.10 |  |  | 1.04 |  |  | 1.90 |  |  | 22.0 |  |  | 1.00 |  |  | 1.00 |  |  | 1.00 |  |  | 1.00 |  |  | 1.00 |  |
|  | s(E_V_T) |  | 1.00 |  |  | 1.00 |  |  | 1.00 |  |  | 1.00 |  |  | 1.00 |  |  | 1.00 |  |  | 1.00 |  |  | 1.03 |  |  | 1.00 |  |  | 1.00 |  |
|  | s(period) |  | 0.75 |  |  | 0.96 |  |  | 0.95 |  |  | 0.99 |  |  | 0.89 |  |  | 0.43 |  |  | 0.77 |  |  | 0.53 |  |  | 0.94 |  |  | 0.81 |  |
|  |  |  |  |  |  |  |  |  |  |  |  |  |  |  |  |  |  |  |  |  |  |  |  |  |  |  |  |  |  |  |  |
| **V_G_** | s(indiv) | 0.77 | 1.00 | * | 0.63 | 1.00 | ** | 0.91 | 1.00 | * | 0.86 | 1.00 | * | 0.52 | 1.00 | *** | 0.88 | 1.00 | * | 0.74 | 1.00 | * | 0.82 | 1.00 | * | 0.97 | 1.00 | * | 0.63 | 1.00 | ** |
|  | s(T_MAX_) |  | 21.0 |  |  | 14.0 |  |  | 23.1 |  |  | 1.00 |  |  | 1.10 |  |  | 1.00 |  |  | 1.00 |  |  | 1.00 |  |  | 1.00 |  |  | 1.00 |  |
|  | s(T_MIN_) |  | 1.00 |  |  | 1.00 |  |  | 1.00 |  |  | 1.00 |  |  | 1.00 |  |  | 1.00 |  |  | 1.00 |  |  | 1.09 |  |  | 1.00 |  |  | 1.00 |  |
|  | s(V_PD_) |  | 1.00 |  |  | 1.00 |  |  | 1.00 |  |  | 1.00 |  |  | 1.00 |  |  | 1.00 |  |  | 1.00 |  |  | 1.00 |  |  | 1.00 |  |  | 1.00 |  |
|  | s(P_RE_) |  | 18.0 |  |  | 11.0 |  |  | 1.00 |  |  | 1.90 |  |  | 0.92 |  |  | 1.00 |  |  | 1.00 |  |  | 1.00 |  |  | 1.00 |  |  | 1.00 |  |
|  | s(E_V_T) |  | 1.00 |  |  | 1.00 |  |  | 1.00 |  |  | 1.00 |  |  | 1.00 |  |  | 1.00 |  |  | 1.00 |  |  | 1.00 |  |  | 1.00 |  |  | 1.00 |  |
|  | s(period) |  | 0.95 |  |  | 0.88 |  |  | 0.88 |  |  | 0.79 |  |  | 0.74 |  |  | 0.57 |  |  | 0.92 |  |  | 0.76 |  |  | 0.73 |  |  | 0.87 |  |
|  |  |  |  |  |  |  |  |  |  |  |  |  |  |  |  |  |  |  |  |  |  |  |  |  |  |  |  |  |  |  |  |
| **V_S_** | s(indiv) | 0.74 | 1.00 | * | 0.61 | 1.00 | ** | 0.88 | 1.00 | * | 0.82 | 1.00 | * | 0.482 | 1.00 | *** | 0.79 | 1.00 | * | 0.68 | 1.00 | ** | 0.78 | 1.00 | * | 0.92 | 1.00 | * | 0.62 | 1.00 | ** |
|  | s(T_MAX_) |  | 19.0 |  |  | 12.0 |  |  | 18.1 |  |  | 1.00 |  |  | 1.00 |  |  | 1.00 |  |  | 1.00 |  |  | 1.00 |  |  | 1.00 |  |  | 1.09 |  |
|  | s(T_MIN_) |  | 1.00 |  |  | 1.00 |  |  | 1.00 |  |  | 1.00 |  |  | 1.00 |  |  | 1.00 |  |  | 1.00 |  |  | 1.00 |  |  | 1.00 |  |  | 1.00 |  |
|  | s(V_PD_) |  | 1.00 |  |  | 1.00 |  |  | 1.00 |  |  | 1.00 |  |  | 1.00 |  |  | 1.00 |  |  | 1.00 |  |  | 1.00 |  |  | 1.00 |  |  | 1.00 |  |
|  | s(P_RE_) |  | 15.0 |  |  | 11.0 |  |  | 1.00 |  |  | 1.00 |  |  | 1.00 |  |  | 1.00 |  |  | 1.00 |  |  | 1.00 |  |  | 1.00 |  |  | 1.00 |  |
|  | s(E_V_T) |  | 1.00 |  |  | 1.00 |  |  | 1.00 |  |  | 1.00 |  |  | 1.00 |  |  | 1.00 |  |  | 1.00 |  |  | 1.00 |  |  | 1.00 |  |  | 1.00 |  |
|  | s(period) |  | 0.90 |  |  | 0.80 |  |  | 0.70 |  |  | 0.66 |  |  | 0.60 |  |  | 0.67 |  |  | 0.76 |  |  | 0.54 |  |  | 0.65 |  |  | 0.75 |  |
|  |  |  |  |  |  |  |  |  |  |  |  |  |  |  |  |  |  |  |  |  |  |  |  |  |  |  |  |  |  |  |  |
| **Fl** | s(indiv) | 0.62 | 1.00 | ** | 0.76 | 1.00 | * | 0.86 | 1.00 | * | 0.95 | 1.00 | * | 0.63 | 1.00 | ** | 0.72 | 1.00 | * | 0.94 | 1.00 | * | 0.82 | 1.00 | * | 0.82 | 1.00 | * | 0.86 | 1.00 | * |
|  | s(T_MAX_) |  | 1.08 |  |  | 1.08 |  |  | 1.10 |  |  | 1.00 |  |  | 1.10 |  |  | 1.00 |  |  | 1.00 |  |  | 1.00 |  |  | 1.00 |  |  | 1.00 |  |
|  | s(T_MIN_) |  | 1.00 |  |  | 1.00 |  |  | 1.00 |  |  | 1.00 |  |  | 1.00 |  |  | 1.00 |  |  | 1.00 |  |  | 1.00 |  |  | 1.00 |  |  | 1.00 |  |
|  | s(V_PD_) |  | 1.00 |  |  | 1.00 |  |  | 1.00 |  |  | 1.00 |  |  | 1.00 |  |  | 1.00 |  |  | 1.00 |  |  | 1.00 |  |  | 1.00 |  |  | 1.00 |  |
|  | s(P_RE_) |  | 21.0 |  |  | 15.0 |  |  | 1.00 |  |  | 1.90 |  |  | 1.00 |  |  | 1.00 |  |  | 1.00 |  |  | 1.00 |  |  | 1.00 |  |  | 1.00 |  |
|  | s(E_V_T) |  | 1.00 |  |  | 1.00 |  |  | 1.00 |  |  | 1.00 |  |  | 1.00 |  |  | 1.00 |  |  | 1.00 |  |  | 1.00 |  |  | 1.00 |  |  | 1.00 |  |
|  | s(period) |  | 0.86 |  |  | 0.70 |  |  | 0.92 |  |  | 0.81 |  |  | 0.82 |  |  | 0.59 |  |  | 0.83 |  |  | 0.46 |  |  | 0.80 |  |  | 0.92 |  |
|  |  |  |  |  |  |  |  |  |  |  |  |  |  |  |  |  |  |  |  |  |  |  |  |  |  |  |  |  |  |  |  |
| **L_UR_** | s(indiv) | 0.77 | 1.00 | * | 0.80 | 1.00 | * | 0.72 | 1.00 | * | 0.82 | 1.00 | * | 0.79 | 1.00 | * | 0.93 | 1.00 | * | 0.79 | 1.00 | * | 0.93 | 1.00 | * | 0.74 | 1.00 | * | 0.80 | 1.00 | * |
|  | s(T_MAX_) |  | 1.01 |  |  | 1.12 |  |  | 1.10 |  |  | 1.00 |  |  | 1.10 |  |  | 1.00 |  |  | 1.00 |  |  | 1.00 |  |  | 1.00 |  |  | 1.00 |  |
|  | s(T_MIN_) |  | 1.00 |  |  | 1.00 |  |  | 1.00 |  |  | 1.00 |  |  | 1.00 |  |  | 1.00 |  |  | 1.00 |  |  | 1.00 |  |  | 1.00 |  |  | 1.00 |  |
|  | s(V_PD_) |  | 1.00 |  |  | 1.00 |  |  | 1.00 |  |  | 1.00 |  |  | 1.00 |  |  | 1.00 |  |  | 1.00 |  |  | 1.00 |  |  | 1.00 |  |  | 1.00 |  |
|  | s(P_RE_) |  | 1.40 |  |  | 1.90 |  |  | 1.00 |  |  | 1.04 |  |  | 1.00 |  |  | 1.00 |  |  | 1.01 |  |  | 1.00 |  |  | 1.07 |  |  | 1.00 |  |
|  | s(E_V_T) |  | 1.00 |  |  | 1.00 |  |  | 1.00 |  |  | 1.00 |  |  | 1.00 |  |  | 1.00 |  |  | 1.00 |  |  | 1.00 |  |  | 1.00 |  |  | 1.00 |  |
|  | s(period) |  | 0.98 |  |  | 0.86 |  |  | 0.86 |  |  | 0.88 |  |  | 0.72 |  |  | 0.77 |  |  | 0.68 |  |  | 0.69 |  |  | 0.77 |  |  | 0.90 |  |
|  |  |  |  |  |  |  |  |  |  |  |  |  |  |  |  |  |  |  |  |  |  |  |  |  |  |  |  |  |  |  |  |
| **W_RY_** | s(indiv) | 0.76 | 1.00 | * | 0.56 | 1.00 | ****** | 0.71 | 1.00 | ***** | **0.42** | 1.00 | ****** | 0.52 | 1.00 | ***** | 0.57 | 1.00 | ***** | 0.56 | 1.00 | * | 0.55 | 1.00 | * | 0.34 | 1.00 | *** | 0.23 | 1.00 | *** |
|  | s(T_MAX_) |  | 1.00 |  |  | 1.00 |  |  | 1.00 |  |  | 1.00 |  |  | 1.00 |  |  | 1.00 |  |  | 1.00 |  |  | 1.00 |  |  | 1.00 |  |  | 1.00 |  |
|  | s(T_MIN_) |  | 1.00 |  |  | 1.00 |  |  | 1.00 |  |  | 1.00 |  |  | 1.00 |  |  | 1.00 |  |  | 1.00 |  |  | 1.00 |  |  | 1.00 |  |  | 1.00 |  |
|  | s(V_PD_) |  | 1.00 |  |  | 1.00 |  |  | 1.00 |  |  | 1.00 |  |  | 1.00 |  |  | 1.00 |  |  | 1.00 |  |  | 1.00 |  |  | 1.00 |  |  | 1.00 |  |
|  | s(P_RE_) |  | 1.00 |  |  | 1.00 |  |  | 1.00 |  |  | 1.00 |  |  | 1.00 |  |  | 1.00 |  |  | 1.00 |  |  | 1.00 |  |  | 1.00 |  |  | 1.00 |  |
|  | s(E_V_T) |  | 1.00 |  |  | 1.10 |  |  | 1.61 |  |  | 1.00 |  |  | 1.00 |  |  | 1.00 |  |  | 1.00 |  |  | 1.00 |  |  | 1.00 |  |  | 1.00 |  |
|  | s(period) |  | 0.98 |  |  | 0.87 |  |  | 0.91 |  |  | 0.81 |  |  | 0.75 |  |  | 0.98 |  |  | 0.83 |  |  | 0.90 |  |  | 0.98 |  |  | 0.78 |  |

|  |  | **Tropical montane cloud forest tree species** | | | | | | | | | | | | | | | | | | | | | | | | | | | | | |
| --- | --- | --- | --- | --- | --- | --- | --- | --- | --- | --- | --- | --- | --- | --- | --- | --- | --- | --- | --- | --- | --- | --- | --- | --- | --- | --- | --- | --- | --- | --- | --- |
|  |  | ***Carya palmeri*** | | | ***Ulmus mexicana*** | | | ***Tilia mexicana*** | | | ***Fagus mexicana*** | | | ***Magnolia schiedeana*** | | | ***Beilchsmiedia mexicana*** | | | ***Styrax glabrescens*** | | | ***Sambucus nigra*** | | | ***Turpinia insignis*** | | | ***Symplocos speciosa*** | | |
| **Leaf morphological traits** | **Factor** | R^2^ | edf | *p* | R^2^ | edf | *p* | R^2^ | edf | *p* | R^2^ | edf | *p* | R^2^ | edf | *p* | R^2^ | edf | *p* | R^2^ | edf | *p* | R^2^ | edf | *p* | R^2^ | edf | *p* | R^2^ | edf | *p* |
| ***S*h** | s(indiv) | 0.54 | 1.00 | * | 0.34 | 1.00 | *** | 0.51 | 1.00 | * | 0.52 | 1.00 | * | 0.55 | 1.00 | * | 0.50 | 1.00 | * | 0.33 | 1.00 | *** | 0.44 | 1.00 | ** | 0.52 | 1.00 | * | 0.49 | 1.00 | ** |
|  | s(T_MAX_) |  | 1.00 |  |  | 1.00 |  |  | 1.00 |  |  | 1.00 |  |  | 1.00 |  |  | 1.00 |  |  | 1.00 |  |  | 1.00 |  |  | 1.00 |  |  | 1.00 |  |
|  | s(T_MIN_) |  | 1.00 |  |  | 1.00 |  |  | 1.00 |  |  | 1.00 |  |  | 1.00 |  |  | 1.87 |  |  | 1.00 |  |  | 1.00 |  |  | 1.00 |  |  | 1.00 |  |
|  | s(V_PD_) |  | 1.00 |  |  | 1.00 |  |  | 1.00 |  |  | 1.00 |  |  | 1.00 |  |  | 1.00 |  |  | 1.00 |  |  | 1.06 |  |  | 1.06 |  |  | 1.06 |  |
|  | s(P_RE_) |  | 1.00 |  |  | 1.00 |  |  | 1.00 |  |  | 1.00 |  |  | 1.00 |  |  | 1.01 |  |  | 1.01 |  |  | 1.01 |  |  | 1.01 |  |  | 1.01 |  |
|  | s(E_V_T) |  | 1.00 |  |  | 1.00 |  |  | 1.00 |  |  | 1.00 |  |  | 1.00 |  |  | 1.00 |  |  | 1.00 |  |  | 1.01 |  |  | 1.01 |  |  | 1.01 |  |
|  | s(period) |  | 0.88 |  |  | 0.80 |  |  | 0.98 |  |  | 0.90 |  |  | 0.77 |  |  | 0.75 |  |  | 0.65 |  |  | 0.76 |  |  | 0.96 |  |  | 0.78 |  |
|  |  |  |  |  |  |  |  |  |  |  |  |  |  |  |  |  |  |  |  |  |  |  |  |  |  |  |  |  |  |  |  |
| ***L*a** | s(indiv) | 0.52 | 1.00 | * | 0.33 | 1.00 | ** | 0.42 | 1.00 | ** | 0.42 | 1.00 | *** | 0.52 | 1.00 | ***** | 0.55 | 1.00 | *** | 0.42 | 1.00 | ** | 0.52 | 1.00 | * | 0.52 | 1.00 | * | 0.47 | 1.00 | ** |
|  | s(T_MAX_) |  | 1.30 |  |  | 1.30 |  |  | 1.30 |  |  | 1.00 |  |  | 1.00 |  |  | 1.00 |  |  | 1.03 |  |  | 1.10 |  |  | 1.00 |  |  | 1.90 |  |
|  | s(T_MIN_) |  | 1.00 |  |  | 1.00 |  |  | 1.00 |  |  | 1.00 |  |  | 1.00 |  |  | 1.00 |  |  | 1.00 |  |  | 1.00 |  |  | 1.00 |  |  | 1.01 |  |
|  | s(V_PD_) |  | 1.00 |  |  | 1.00 |  |  | 1.00 |  |  | 1.00 |  |  | 1.00 |  |  | 1.00 |  |  | 1.01 |  |  | 1.10 |  |  | 1.00 |  |  | 1.00 |  |
|  | s(P_RE_) |  | 1.00 |  |  | 1.00 |  |  | 1.00 |  |  | 1.10 |  |  | 1.00 |  |  | 1.00 |  |  | 1.00 |  |  | 1.00 |  |  | 1.00 |  |  | 1.00 |  |
|  | s(E_V_T) |  | 1.00 |  |  | 1.00 |  |  | 1.00 |  |  | 1.00 |  |  | 1.00 |  |  | 1.00 |  |  | 1.01 |  |  | 1.09 |  |  | 1.00 |  |  | 1.04 |  |
|  | s(period) |  | 0.92 |  |  | 0.78 |  |  | 0.86 |  |  | 0.56 |  |  | 0.67 |  |  | 0.73 |  |  | 0.70 |  |  | 0.88 |  |  | 0.98 |  |  | 0.79 |  |
|  |  |  |  |  |  |  |  |  |  |  |  |  |  |  |  |  |  |  |  |  |  |  |  |  |  |  |  |  |  |  |  |
| ***L*o** | s(indiv) | 0.48 | 1.00 | * | 0.30 | 1.00 | ** | 0.39 | 1.00 | ** | 0.40 | 1.00 | *** | 0.48 | 1.00 | ***** | 0.50 | 1.00 | ** | 0.45 | 1.00 | ** | 0.33 | 1.00 | *** | 0.48 | 1.00 | ** | 0.40 | 1.00 | ** |
|  | s(T_MAX_) |  | 1.09 |  |  | 1.30 |  |  | 1.09 |  |  | 1.00 |  |  | 1.00 |  |  | 1.00 |  |  | 1.22 |  |  | 1.00 |  |  | 1.00 |  |  | 1.90 |  |
|  | s(T_MIN_) |  | 1.00 |  |  | 1.00 |  |  | 1.00 |  |  | 1.00 |  |  | 1.00 |  |  | 1.00 |  |  | 1.00 |  |  | 1.00 |  |  | 1.00 |  |  | 1.10 |  |
|  | s(V_PD_) |  | 1.00 |  |  | 1.00 |  |  | 1.00 |  |  | 1.00 |  |  | 1.00 |  |  | 1.00 |  |  | 1.00 |  |  | 1.10 |  |  | 1.00 |  |  | 1.00 |  |
|  | s(P_RE_) |  | 1.00 |  |  | 1.00 |  |  | 1.00 |  |  | 1.01 |  |  | 1.00 |  |  | 1.00 |  |  | 1.00 |  |  | 1.00 |  |  | 1.00 |  |  | 1.00 |  |
|  | s(E_V_T) |  | 1.00 |  |  | 1.00 |  |  | 1.00 |  |  | 1.00 |  |  | 1.00 |  |  | 1.00 |  |  | 1.00 |  |  | 1.00 |  |  | 1.00 |  |  | 1.00 |  |
|  | s(period) |  | 0.78 |  |  | 0.70 |  |  | 0.83 |  |  | 0.50 |  |  | 0.62 |  |  | 0.70 |  |  | 0.74 |  |  | 0.65 |  |  | 0.78 |  |  | 0.68 |  |
|  |  |  |  |  |  |  |  |  |  |  |  |  |  |  |  |  |  |  |  |  |  |  |  |  |  |  |  |  |  |  |  |
| ***L*l** | s(indiv) | 0.59 | 1.00 | ** | 0.68 | 1.00 | * | 0.73 | 1.00 | * | 0.72 | 1.00 | * | 0.49 | 1.00 | ** | 0.80 | 1.00 | * | 0.50 | 1.00 | * | 0.72 | 1.00 | * | 0.78 | 1.00 | * | 0.69 | 1.00 | * |
|  | s(T_MAX_) |  | 1.00 |  |  | 1.00 |  |  | 1.09 |  |  | 1.00 |  |  | 1-00 |  |  | 1.00 |  |  | 1.00 |  |  | 1.00 |  |  | 1.40 |  |  | 1.00 |  |
|  | s(T_MIN_) |  | 1.00 |  |  | 1.00 |  |  | 1.01 |  |  | 1.00 |  |  | 1.00 |  |  | 1.00 |  |  | 1.00 |  |  | 12.0 |  |  | 1.00 |  |  | 1.00 |  |
|  | s(V_PD_) |  | 1.00 |  |  | 1.00 |  |  | 1.00 |  |  | 2.00 |  |  | 1.09 |  |  | 1.00 |  |  | 1.00 |  |  | 1.00 |  |  | 1.00 |  |  | 1.00 |  |
|  | s(P_RE_) |  | 1.00 |  |  | 1.10 |  |  | 1.01 |  |  | 3.00 |  |  | 1.00 |  |  | 1.00 |  |  | 1.00 |  |  | 1.04 |  |  | 1.00 |  |  | 1.00 |  |
|  | s(E_V_T) |  | 1.00 |  |  | 1.00 |  |  | 1.00 |  |  | 1.00 |  |  | 1.00 |  |  | 1.00 |  |  | 1.02 |  |  | 1.01 |  |  | 1.00 |  |  | 1.03 |  |
|  | s(period) |  | 0.72 |  |  | 0.84 |  |  | 0.89 |  |  | 0.86 |  |  | 0.79 |  |  | 0.62 |  |  | 0.68 |  |  | 0.56 |  |  | 0.82 |  |  | 0.78 |  |
|  |  |  |  |  |  |  |  |  |  |  |  |  |  |  |  |  |  |  |  |  |  |  |  |  |  |  |  |  |  |  |  |
| ***A*l** | s(indiv) | 0.72 | 1.00 | * | 0.84 | 1.00 | * | 0.23 | 1.00 | *** | 0.80 | 1.00 | * | 0.70 | 1.00 | * | 0.78 | 1.00 | * | 0.86 | 1.00 | * | 0.54 | 1.00 | * | 0.77 | 1.00 | * | 0.63 | 1.00 | * |
|  | s(T_MAX_) |  | 20.0 |  |  | 1.00 |  |  | 1.00 |  |  | 1.00 |  |  | 1.00 |  |  | 1.00 |  |  | 1.00 |  |  | 1.00 |  |  | 1.40 |  |  | 1.00 |  |
|  | s(T_MIN_) |  | 1.00 |  |  | 1.00 |  |  | 1.00 |  |  | 1.00 |  |  | 1.00 |  |  | 1.09 |  |  | 1.00 |  |  | 1.00 |  |  | 1.00 |  |  | 1.00 |  |
|  | s(V_PD_) |  | 1.00 |  |  | 1.00 |  |  | 1.00 |  |  | 1.00 |  |  | 1.00 |  |  | 1.00 |  |  | 1.00 |  |  | 1.00 |  |  | 1.00 |  |  | 1.00 |  |
|  | s(P_RE_) |  | 1.00 |  |  | 1.00 |  |  | 1.00 |  |  | 1.00 |  |  | 1.00 |  |  | 1.00 |  |  | 1.00 |  |  | 1.00 |  |  | 1.00 |  |  | 1.00 |  |
|  | s(E_V_T) |  | 1.19 |  |  | 1.00 |  |  | 1.00 |  |  | 1.00 |  |  | 1.00 |  |  | 1.00 |  |  | 1.00 |  |  | 1.00 |  |  | 1.00 |  |  | 1.03 |  |
|  | s(period) |  | 0.88 |  |  | 0.77 |  |  | 0.68 |  |  | 0.67 |  |  | 0.86 |  |  | 0.89 |  |  | 0.86 |  |  | 0.90 |  |  | 0.75 |  |  | 0.72 |  |
|  |  |  |  |  |  |  |  |  |  |  |  |  |  |  |  |  |  |  |  |  |  |  |  |  |  |  |  |  |  |  |  |
| ***A*a** | s(indiv) | 0.55 | 1.00 | * | 0.35 | 1.00 | *** | 0.49 | 1.00 | ** | 0.65 | 1.00 | * | 0.77 | 1.00 | * | 0.80 | 1.00 | * | 0.45 | 1.00 | ** | 0.55 | 1.00 | * | 0.69 | 1.00 | * | 0.32 | 1.00 | *** |
|  | s(T_MAX_) |  | 10.0 |  |  | 1.00 |  |  | 1.00 |  |  | 1.10 |  |  | 1.00 |  |  | 1.00 |  |  | 1.00 |  |  | 1.00 |  |  | 24.0 |  |  | 1.00 |  |
|  | s(T_MIN_) |  | 1.00 |  |  | 1.00 |  |  | 1.00 |  |  | 1.00 |  |  | 1.00 |  |  | 1.09 |  |  | 1.00 |  |  | 1.00 |  |  | 1.00 |  |  | 1.00 |  |
|  | s(V_PD_) |  | 1.00 |  |  | 1.00 |  |  | 1.00 |  |  | 1.00 |  |  | 1.00 |  |  | 1.00 |  |  | 1.00 |  |  | 1.00 |  |  | 1.00 |  |  | 1.00 |  |
|  | s(P_RE_) |  | 1.00 |  |  | 1.00 |  |  | 1.00 |  |  | 1.00 |  |  | 1.00 |  |  | 1.00 |  |  | 1.00 |  |  | 1.00 |  |  | 1.06 |  |  | 1.00 |  |
|  | s(E_V_T) |  | 1.00 |  |  | 1.00 |  |  | 1.00 |  |  | 1.00 |  |  | 1.00 |  |  | 1.00 |  |  | 1.00 |  |  | 1.00 |  |  | 1.00 |  |  | 1.00 |  |
|  | s(period) |  | 0.90 |  |  | 0.43 |  |  | 0.56 |  |  | 0.88 |  |  | 0.78 |  |  | 0.92 |  |  | 0.69 |  |  | 0.95 |  |  | 0.91 |  |  | 0.34 |  |
|  |  |  |  |  |  |  |  |  |  |  |  |  |  |  |  |  |  |  |  |  |  |  |  |  |  |  |  |  |  |  |  |
| ***A*s** | s(indiv) | 0.59 | 1.00 | * | 0.69 | 1.00 | * | 0.66 | 1.00 | * | 0.87 | 1.00 | * | 0.43 | 1.00 | ** | 0.85 | 1.00 | * | 0.72 | 1.00 | * | 0.45 | 1.00 | ** | 0.80 | 1.00 | * | 0.68 | 1.00 | * |
|  | s(T_MAX_) |  | 1.00 |  |  | 12.0 |  |  | 1.00 |  |  | 1.00 |  |  | 10.1 |  |  | 1.00 |  |  | 1.00 |  |  | 1.00 |  |  | 1.00 |  |  | 1.00 |  |
|  | s(T_MIN_) |  | 1.00 |  |  | 1.00 |  |  | 1.00 |  |  | 1.00 |  |  | 1.00 |  |  | 1.00 |  |  | 1.00 |  |  | 1.09 |  |  | 1.00 |  |  | 1.00 |  |
|  | s(V_PD_) |  | 1.00 |  |  | 1.00 |  |  | 1.00 |  |  | 1.00 |  |  | 1.00 |  |  | 1.00 |  |  | 1.00 |  |  | 1.00 |  |  | 1.00 |  |  | 1.00 |  |
|  | s(P_RE_) |  | 1.00 |  |  | 1.00 |  |  | 1.00 |  |  | 1.00 |  |  | 12.0 |  |  | 1.00 |  |  | 1.00 |  |  | 1.00 |  |  | 1.00 |  |  | 1.00 |  |
|  | s(E_V_T) |  | 1.00 |  |  | 1.00 |  |  | 1.00 |  |  | 1.00 |  |  | 1.00 |  |  | 1.00 |  |  | 1.00 |  |  | 1.01 |  |  | 1.00 |  |  | 1.00 |  |
|  | s(period) |  | 0.84 |  |  | 0.89 |  |  | 0.85 |  |  | 0.99 |  |  | 0.89 |  |  | 0.73 |  |  | 0.83 |  |  | 0.75 |  |  | 0.80 |  |  | 0.89 |  |
|  |  |  |  |  |  |  |  |  |  |  |  |  |  |  |  |  |  |  |  |  |  |  |  |  |  |  |  |  |  |  |  |
| ***B*a** | s(indiv) | 0.64 | 1.00 | ** | 0.73 | 1.00 | * | 0.84 | 1.00 | * | 0.90 | 1.00 | * | 0.59 | 1.00 | * | 0.34 | 1.00 | *** | 0.33 | 1.00 | *** | 0.78 | 1.00 | * | 0.44 | 1.00 | ** | 0.88 | 1.00 | * |
|  | s(T_MAX_) |  | 1.08 |  |  | 1.01 |  |  | 1.10 |  |  | 1.00 |  |  | 1.09 |  |  | 1.00 |  |  | 1.00 |  |  | 1.00 |  |  | 1.00 |  |  | 1.09 |  |
|  | s(T_MIN_) |  | 1.00 |  |  | 1.00 |  |  | 1.00 |  |  | 1.00 |  |  | 1.00 |  |  | 1.00 |  |  | 1.00 |  |  | 1.00 |  |  | 1.00 |  |  | 1.00 |  |
|  | s(V_PD_) |  | 1.00 |  |  | 1.00 |  |  | 1.00 |  |  | 1.00 |  |  | 1.00 |  |  | 1.00 |  |  | 1.00 |  |  | 1.00 |  |  | 1.00 |  |  | 1.00 |  |
|  | s(P_RE_) |  | 1.09 |  |  | 23.0 |  |  | 1.00 |  |  | 1.94 |  |  | 1.00 |  |  | 1.00 |  |  | 1.00 |  |  | 1.00 |  |  | 1.00 |  |  | 1.00 |  |
|  | s(E_V_T) |  | 1.00 |  |  | 1.00 |  |  | 1.00 |  |  | 1.00 |  |  | 1.00 |  |  | 1.00 |  |  | 1.00 |  |  | 1.00 |  |  | 1.00 |  |  | 1.00 |  |
|  | s(period) |  | 0.85 |  |  | 0.76 |  |  | 0.87 |  |  | 0.78 |  |  | 0.78 |  |  | 0.63 |  |  | 0.75 |  |  | 0.54 |  |  | 0.77 |  |  | 0.84 |  |
|  |  |  |  |  |  |  |  |  |  |  |  |  |  |  |  |  |  |  |  |  |  |  |  |  |  |  |  |  |  |  |  |
| ***N*b** | s(indiv) | 0.55 | 1.00 | * | 0.77 | 1.00 | * | 0.45 | 1.00 | ** | 0.53 | 1.00 | * | 0.66 | 1.00 | * | 0.33 | 1.00 | *** | 0.30 | 1.00 | *** | 0.72 | 1.00 | * | 0.34 | 1.00 | *** | 0.48 | 1.00 | ** |
|  | s(T_MAX_) |  | 34.0 |  |  | 1.00 |  |  | 1.00 |  |  | 31.0 |  |  | 1.09 |  |  | 1.00 |  |  | 1.00 |  |  | 1.00 |  |  | 1.00 |  |  | 1.12 |  |
|  | s(T_MIN_) |  | 1.00 |  |  | 1.00 |  |  | 1.00 |  |  | 1.00 |  |  | 1.00 |  |  | 1.00 |  |  | 1.00 |  |  | 1.00 |  |  | 1.00 |  |  | 1.00 |  |
|  | s(V_PD_) |  | 1.00 |  |  | 1.00 |  |  | 1.00 |  |  | 1.00 |  |  | 1.00 |  |  | 1.00 |  |  | 1.00 |  |  | 1.00 |  |  | 1.00 |  |  | 1.00 |  |
|  | s(P_RE_) |  | 1.00 |  |  | 1.00 |  |  | 1.00 |  |  | 1.21 |  |  | 1.00 |  |  | 1.00 |  |  | 1.00 |  |  | 1.00 |  |  | 1.00 |  |  | 1.00 |  |
|  | s(E_V_T) |  | 1.00 |  |  | 1.00 |  |  | 1.00 |  |  | 1.00 |  |  | 1.00 |  |  | 1.00 |  |  | 1.00 |  |  | 1.10 |  |  | 1.00 |  |  | 1.00 |  |
|  | s(period) |  | 0.96 |  |  | 0.88 |  |  | 0.76 |  |  | 0.89 |  |  | 0.83 |  |  | 0.56 |  |  | 0.63 |  |  | 0.63 |  |  | 0.87 |  |  | 0.69 |  |
|  |  |  |  |  |  |  |  |  |  |  |  |  |  |  |  |  |  |  |  |  |  |  |  |  |  |  |  |  |  |  |  |
| ***V*d** | s(indiv) | 0.59 | 1.00 | * | 0.68 | 1.00 | * | 0.70 | 1.00 | * | 0.69 | 1.00 | * | 0.23 | 1.00 | *** | 0.22 | 1.00 | *** | 0.67 | 1.00 | * | 0.69 | 1.00 | * | 0.80 | 1.00 | * | 0.68 | 1.00 | * |
|  | s(T_MAX_) |  | 1.00 |  |  | 1.00 |  |  | 1.10 |  |  | 1.00 |  |  | 1.00 |  |  | 1.00 |  |  | 1.00 |  |  | 1.00 |  |  | 1.00 |  |  | 1.00 |  |
|  | s(T_MIN_) |  | 1.00 |  |  | 1.00 |  |  | 1.00 |  |  | 1.00 |  |  | 1.00 |  |  | 1.00 |  |  | 1.00 |  |  | 3.00 |  |  | 1.00 |  |  | 1.00 |  |
|  | s(V_PD_) |  | 1.00 |  |  | 1.00 |  |  | 1.00 |  |  | 1.04 |  |  | 1.00 |  |  | 1.00 |  |  | 1.00 |  |  | 1.00 |  |  | 1.00 |  |  | 1.00 |  |
|  | s(P_RE_) |  | 1.00 |  |  | 1.10 |  |  | 1.00 |  |  | 1.00 |  |  | 2.08 |  |  | 1.00 |  |  | 1.00 |  |  | 1.00 |  |  | 1.00 |  |  | 1.00 |  |
|  | s(E_V_T) |  | 1.00 |  |  | 1.00 |  |  | 1.00 |  |  | 1.00 |  |  | 1.00 |  |  | 1.00 |  |  | 1.00 |  |  | 1.00 |  |  | 1.00 |  |  | 1.00 |  |
|  | s(period) |  | 0.72 |  |  | 0.89 |  |  | 0.90 |  |  | 0.88 |  |  | 0.80 |  |  | 0.40 |  |  | 0.68 |  |  | 0.43 |  |  | 0.90 |  |  | 0.78 |  |
|  |  |  |  |  |  |  |  |  |  |  |  |  |  |  |  |  |  |  |  |  |  |  |  |  |  |  |  |  |  |  |  |
| ***A*v** | s(indiv) | 0.74 | 1.00 | * | 0.57 | 1.00 | ***** | 0.33 | 1.00 | ******* | **0.55** | 1.00 | ***** | 0.49 | 1.00 | ****** | 0.44 | 1.00 | ****** | 0.33 | 1.00 | *** | 0.23 | 1.00 | *** | 0.33 | 1.00 | *** | 0.22 | 1.00 | *** |
|  | s(T_MAX_) |  | 1.00 |  |  | 1.00 |  |  | 1.00 |  |  | 1.00 |  |  | 1.00 |  |  | 1.00 |  |  | 1.00 |  |  | 1.00 |  |  | 1.00 |  |  | 1.00 |  |
|  | s(T_MIN_) |  | 1.00 |  |  | 1.00 |  |  | 1.00 |  |  | 1.00 |  |  | 1.00 |  |  | 1.00 |  |  | 1.00 |  |  | 1.00 |  |  | 1.00 |  |  | 1.00 |  |
|  | s(V_PD_) |  | 1.00 |  |  | 1.00 |  |  | 1.00 |  |  | 1.00 |  |  | 1.00 |  |  | 1.00 |  |  | 1.00 |  |  | 1.00 |  |  | 1.00 |  |  | 1.00 |  |
|  | s(P_RE_) |  | 1.00 |  |  | 1.00 |  |  | 1.00 |  |  | 1.00 |  |  | 1.00 |  |  | 1.00 |  |  | 1.00 |  |  | 1.00 |  |  | 1.00 |  |  | 1.00 |  |
|  | s(E_V_T) |  | 1.00 |  |  | 1.10 |  |  | 1.10 |  |  | 1.00 |  |  | 1.00 |  |  | 1.00 |  |  | 1.00 |  |  | 1.00 |  |  | 1.00 |  |  | 1.00 |  |
|  | s(period) |  | 0.91 |  |  | 0.47 |  |  | 0.64 |  |  | 0.79 |  |  | 0.65 |  |  | 0.78 |  |  | 0.71 |  |  | 0.67 |  |  | 0.65 |  |  | 0.58 |  |
|  |  |  |  |  |  |  |  |  |  |  |  |  |  |  |  |  |  |  |  |  |  |  |  |  |  |  |  |  |  |  |  |
| ***N*t** | s(indiv) | 0.55 | 1.00 | * | 0.50 | 1.00 | ***** | 0.53 | 1.00 | ***** | 0.33 | 1.00 | ******* | 0.53 | 1.00 | ***** | 0.32 | 1.00 | ****** | 0.33 | 1.00 | *** | 0.20 | 1.00 | *** | 0.49 | 1.00 | ** | 0.18 | 1.00 | *** |
|  | s(T_MAX_) |  | 1.00 |  |  | 1.00 |  |  | 1.00 |  |  | 1.00 |  |  | 1.00 |  |  | 1.00 |  |  | 1.00 |  |  | 1.00 |  |  | 1.00 |  |  | 1.00 |  |
|  | s(T_MIN_) |  | 1.00 |  |  | 1.00 |  |  | 1.00 |  |  | 1.00 |  |  | 1.00 |  |  | 1.00 |  |  | 1.00 |  |  | 1.00 |  |  | 1.00 |  |  | 1.00 |  |
|  | s(V_PD_) |  | 1.00 |  |  | 1.00 |  |  | 1.00 |  |  | 1.00 |  |  | 1.00 |  |  | 1.00 |  |  | 1.00 |  |  | 1.00 |  |  | 1.00 |  |  | 1.00 |  |
|  | s(P_RE_) |  | 1.00 |  |  | 1.00 |  |  | 1.00 |  |  | 1.00 |  |  | 1.00 |  |  | 1.00 |  |  | 1.00 |  |  | 1.00 |  |  | 1.00 |  |  | 1.00 |  |
|  | s(E_V_T) |  | 1.00 |  |  | 1.10 |  |  | 1.10 |  |  | 1.00 |  |  | 1.00 |  |  | 1.00 |  |  | 1.00 |  |  | 1.00 |  |  | 1.00 |  |  | 1.00 |  |
|  | s(period) |  | 0.87 |  |  | 0.85 |  |  | 0.84 |  |  | 0.65 |  |  | 0.95 |  |  | 0.62 |  |  | 0.61 |  |  | 0.45 |  |  | 075 |  |  | 0.34 |  |

Signif. codes: 0 <= '***' < 0.001 < '**' < 0.01 < '*' < 0.05

**Supplementary Table 2.** Statistical summary of the Generalized Additive Model (GAM) selected, examining the relationship between climatic factors and Tropical montane cloud forest tree species. The table includes the following columns: Factor (climatic variable), R^2^ (adjusted correlation coefficient), edf (effective degrees of freedom), and *p*-value.
